# Protective mechanism of *Erigeron breviscapus* injection on blood–brain barrier injury induced by cerebral ischemia in rats

**DOI:** 10.1038/s41598-021-97908-x

**Published:** 2021-09-16

**Authors:** Guangli Liu, Yan Liang, Min Xu, Ming Sun, Weijun Sun, You Zhou, Xiaojuan Huang, Wenjie Song, Yuan Liang, Zhang Wang

**Affiliations:** 1 College of Pharmacy, Chengdu University of Traditional Chinese Medicine, Chengdu, 611137 Sichuan China; 2 Hospital Pharmaceutical Department, Xuzhou Maternity and Child Health Care Hospital, Xuzhou, 221000 Jiangsu China; 3 College of Ethnomedicine, Chengdu University of Traditional Chinese Medicine, Chengdu, 611137 Sichuan China

**Keywords:** Medicinal chemistry, Neuroscience, Diseases

## Abstract

This study investigates the protective effect of *Erigeron breviscapus* injection, a classic traditional Chinese medicine most typically used by Chinese minority to treat stroke, on cerebral ischemia–reperfusion injury and the related signaling pathways. Use network pharmacology methods to study the relationship between *E. breviscapus* (Vant.) Hand-Mazz. and ischemic stroke, predict the mechanism and active ingredients of *E. breviscapus* (Vant.) Hand-Mazz. in improving ischemic stroke disease. We study the protective effect of *E. breviscapus* injection on blood–brain barrier (BBB) injuries induced by cerebral ischemia in rats by regulating the ROS/RNS-MMPs-TJs signaling pathway. The rat model of focal cerebral ischemia–reperfusion injury has been prepared using the wire-suppository method. Firstly, the efficacy of *E. breviscapus* injection, Scutellarin and 3,5-dicaffeoylquinic acid in protecting BBB injury caused by cerebral ischemia has been evaluated. Secondly, the following two methods have been used to study the mechanism of *E. breviscapus* injection in regulating the ROS/RNS-MMPS-TJS signaling pathway: real-time PCR and western blot for the determination of iNOS, MMP-9, claudin-5, occludin, ZO-1 mRNA and protein expression in brain tissue. We find that PI3K-Akt signaling pathway predicted by network pharmaology affects the blood–brain barrier function, so we chose the blood–brain barrier-related MMP-9, claudin-5, iNOS, occludin and ZO-1 proteins are used for research. The results of our research show that 3 drugs can reduce the rate of cerebral infarction in rats, relieve the abnormal neuroethology of rats, reduce the degree of brain tissue lesion, increase the number of the Nissl corpuscle cells and repair the neuron ultrastructure in injured rats. At the same time, it can obviously reduce the ultrastructure damage of the BBB in rats. All three drugs significantly reduced the content of Evans blue in the ischemic brain tissue caused by cerebral ischemia in rats with BBB injury. In addition, *E. breviscapus* injection, Scutellarin and 3,5-dicaffeoylquinic acid can decrease the protein expression of iNOS and MMP-9 in rat ischemic brain tissue. In addition, 3,5-dicaffeoylquinic acid can increase the protein expression of claudin-5. We conclude that *E. breviscapus* injection, Scutellarin and 3,5-dicaffeoylquinic acid have obvious therapeutic effects on BBB and neuron injury induced by cerebral ischemia in rats. Our results from studying the mechanism of action show that *E. breviscapus* injection and Scutellarin inhibited the activation of MMP-9 by inhibiting the synthesis of iNOS, 3,5-dicaffeoylquinic acid inhibits the expression and activation of MMP-9 by inhibiting the activation of iNOS and reducing the generation of free radicals, thus reducing the degradation of important cytoskeleton connexin claudin-5 in the tight junction (TJ) structure by inhibiting the expression and activation of MMP-9. Finally BBB structure integrity was protected.

## Introduction

Cerebral infarction, also known as ischemia cerebral vascular disease, is a blood supply disorder in local brain tissue that causes irreversible damage to brain tissue due to ischemia and hypoxia. China is a high-incidence area^1^ for atherothrombotic cerebral infarction, cerebral embolism, brain watershed infarction, hemorrhagic infarction, etc^2^. Cerebral ischemia reperfusion injury refers to brain cell damage caused by cerebral ischemia. After the reperfusion of blood, the ischemic injury is further aggravated^3^. The obvious change is neuronal damage and brain edema, which are closely related to blood–brain barrier (BBB) damage.

**Figure 1:**
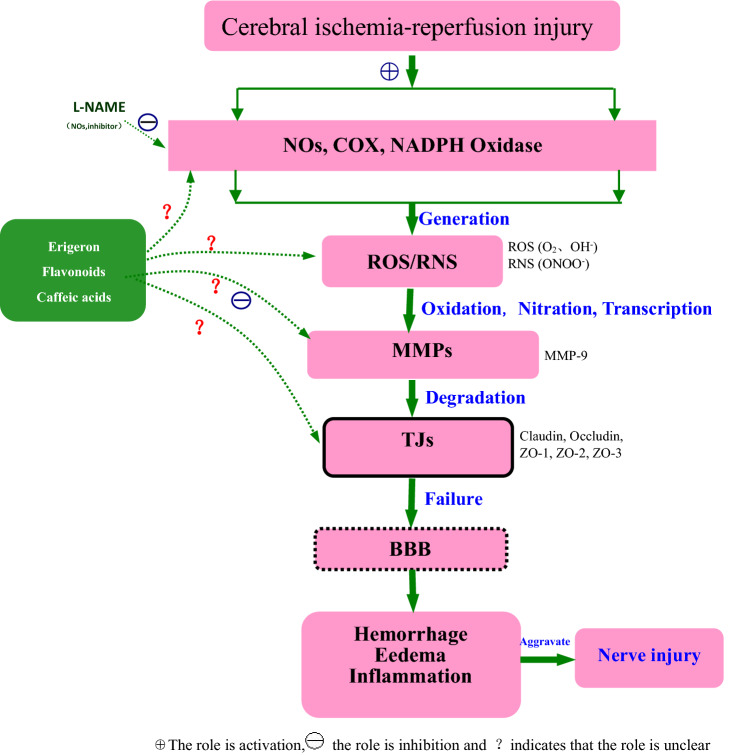
Schematic diagram of ROS/RNS-MMPs-TJs involved in cerebral ischemic BBB injury.

The role of the BBB is to control the transport of certain substances in blood circulation to the central nervous tissue to achieve stability in protecting the environment of the central nervous system. It is a complex cellular system existing between brain tissue and blood, and its permeability is closely related to the degeneration, damage and inflammation of the central nervous system^4^. The BBB consists of three barriers, the first barrier being the vascular endothelial cells without fenestration and the tight junctions between the cells. The junction complex between endothelial cells is composed of a TJ and an adhesive junction^5^. TJ is the structural basis of the BBB, including membrane intrinsic proteins and cytoplasmic accessory proteins, while membrane intrinsic proteins are composed of claudin, occludin and connective adhesion molecules, and cytoplasmic accessory proteins are blocked by small band proteins (i.e., Zonulae Occludens protein, ZO-1, ZO-2 and ZO-3)^6^, cingulate protein and other components. The second BBB includes the basement membrane of extracellular junction and the enzyme barrier composed of nucleotidase and non-specific cholinesterase; the main substances contained are type IV collagen, laminin, fibronectin and so on. The third BBB is the glial membrane barrier that is formed by the basement membrane of the astrocyte glial cells around the blood vessels. The results of our research show that the structural changes of the BBB after ischemia–reperfusion injury were manifested in microvascular endothelial cells, astrocyte foot processes and basement membrane changes, and that the functional abnormalities showed changes in BBB permeability. BBB injury is considered to be an important cause of cerebral ischemia secondary to edema, hemorrhage, inflammation, etc. (Fig. 1).

*The Pharmacopoeia of the People's Republic of China* (2015 edition, Part One) records that breviscapine belongs to the chrysanthemum plant short scapes pterygium *E. breviscapus* (Vant.) Hand-Mazz.. The medicinal site is all-grass^7^. *E. breviscapus* is a common medicine of eight ethnic minorities, such as the Miao, Naxi and Yi nationalities^8^. It has a slightly bitter taste. For the treatment of heart and liver diseases, it boasts the effect of dispelling wind and cold, activating blood circulation, and relieving pain. *Erigeron breviscapus* preparation has become a commonly used drug in the treatment of cerebral ischemia, and has been widely promoted and applied. *Erigeron breviscapus* injection is contained in *the Pharmacopoeia of the People's Republic of China* (2015 edition, Part One), which is a Chinese herbal injection made from the dry whole grass of breviscapine that mainly contains flavonoids and coffee acid compounds. Its main quality control indexes are Scutellarin (C_21_H_18_O_12_, Breviscapine) and 1,3-dicaffeoylquinic acid (C_25_H_24_O_12_). Its main effects are activating blood circulation and relieving pain^7^.

The mechanisms involved in the treatment of ischemic stroke include vasodilation, microcirculation improvement, anti-blood coagulation, free radical scavenging capacity enhancement, reduction of BBB damage, increased anti-brain water content, anti-neuron damage^8^, anti-inflammatory factors and many other aspects. BBB injury mechanisms include ROS/RNS injury^9^, MMPs activation^10^, inflammatory cell infiltration^11^, open water channel^12^ and calcium overload^13^. The common complication of cerebral ischemia–reperfusion injury is vasculogenic brain edema, which is mainly caused by the increase of BBB permeability. Cerebrovascular diseases are caused by abnormal energy metabolism, and the oxidative stress caused by ROS/RNS in blood brain barrier is an important factor. When cerebral ischemia occurs, mitochondrial oxidative phosphorylation stops, and coenzyme I oxidized type and coenzyme I reduced type cannot be regenerated, resulting in obstacles in the aerobic oxidation process. After external stimulation of hypoxia, excessive NO is produced, and reactive oxygen species and NO combine to produce active nitrogen, leading to ROS/RNS damage. The specific process is that the increase of BBB permeability leads to the spillover of plasma protein and water in capillaries, which leads to the increase of extracellular fluid content in brain tissue^14^ eventually causing brain edema. Vascular brain edema is also the main cause of cerebral hernia. In vitro experiments on rat cerebral cortical neurons have shown that several chemical constituents of *E. breviscapus* could improve the survival rate of neurons^15–18^, such as 3,5-dicaffeoylquinic acid, 4,5-dicaffeoylquinic acid, 1,5-dicaffeoylquinic acid, 3,4-dicaffeoylquinic acid, caffeic acid, Scutellarin, apigenin, apigenin-O-7-β-d-glucoside and 1,5-dicaffeoylquinic acid. Acids (i.e., 1,3-dicaffeoyl quinic acid, 3,5-dicaffeoyl quinic acid and 4,5-dicaffeoyl quinic acid) can penetrate the BBB model^19^ in vitro to different degrees and are used to treat BBB injury. *Erigeron breviscapus* injection, as a traditional Chinese medicine injection extracted from *E. breviscapus*, mainly contains Scutellarin (C_21_H_18_O_12_) and total caffeic acid ester, which has been widely recognized and promoted in clinical practice. In vitro experiments on rat cerebral cortical neurons showed that several chemical constituents of *E. breviscapus* could improve the survival rate of neurons^17^, such as 3,5-dicaffeoylquinic acid, 4,5-dicaffeoylquinic acid, 1,5-dicaffeoylquinic acid and 3,4-dicaffeoylquinic acid, caffeic acid, Scutellarin, apigenin and apigenin. O-7-beta-d-glucose, etc. 1,5-dicaffeoylquinic acid, 1,3-dicaffeoylquinic acid, 3,5-dicaffeoylquinic acid and 4,5-dicaffeoylquinic acid can penetrate the BBB model^16^ in vitro to different extents to treat BBB injury.

Clinical studies suggest that *E. breviscapus* injection^18–22^ can significantly reduce serum MMP-9 levels in patients with acute cerebral infarction. At the same time, experimental results show that Erigeron^23^ and Breviscapine^24,25^ could protect the BBB mainly by reducing the expression level of MMP-9 in brain tissue, increasing the expression level of protein, mRNA of ZO-1, claudin-5 and mRNA^26^. In vitro cell experiments show that breviscapine combined with borneol^27^ have a protective effect on BBB injury under hypoxia. The mechanism may be related to the up-regulation of ZO-1 and claudin-5 protein expression. However, based on animal models combined with the BBB injury mechanism, systematic and in-depth studies on the chemical constituents of *E. breviscapus* injection to protect BBB injury caused by cerebral ischemia have been only infrequently reported. In order to fill this gap in the current literature, our paper researches this topic based on the MCAO animal model to study the protective effect of *E. breviscapus* injection and its chemical constituents on BBB injury caused by cerebral ischemia.

In summary, this study takes *E. breviscapus* injection as the research object and BBB protection as the breakthrough point combined with molecular biology experimental methods, to investigate the BBB protective effect of *E. breviscapus* injection, its flavonoids and caffeoyl components on MCAO model rats including whether they participate in regulating the ROS/RNS-MMPs-TJs signaling pathway. The purpose of this study is to elucidate the correlation between *E. breviscapus* injection and the ROS/RNS-MMPs-TJs pathway in order to reveal the modern scientific connotation of *E. breviscapus* injection in the treatment of left paralysis and right paralysis and to provide the basis for effective and rational in-clinic drug use. Therefore, this study has important scientific significance as well as academic and practical value.

## Materials and methods

### Database and analysis software, drugs and reagents

Use TCMSP (http://tcmspw.com/tcmsp.php) to find drug targets; use UniProt (http://www.Uniprot.org) for protein name correction; use Genecards (http://www.genecards.org) for disease target prediction; use String (http://String-db.org) to construct protein interaction maps; use Venny2.1 (http://bionfogp.cnb.csic.es/tools/Venny/) draw a common potential target map of drug diseases; use Cytoscape to draw a drug-component-target-disease network map.

HPLC (High performance liquid chromatography) system is produced by Shimadzu Corporation of Japan, the model is LC-2010A HT. Electronic scales is produced bu Sartorius Corporation of Germany, the modle is BP121S.

*E. breviscapus* injection is produced by Yunnan Biological Valley Pharmaceutical Co., Ltd.; the batch number is 21411131. The daily dose for an adult selected in this study was 60 mL/60 kg/day, 1 mL/kg/day. Therefore, the rat dose was 5 mL/kg/day, which is equivalent to five times the clinical daily dose for adult use^21,22,28–42^. Nimodipine injection is produced by Bayer Medical and Health Co., Ltd. in Beijing; the batch number is BXGHE21. The daily dose for an adult selected in this study was 6 mg/60 kg/day, or 0.1 mg/kg/day. Therefore, the rat dose was 0.5 mg/kg/day, which is equivalent to five times the clinical daily dose for adult use^43^. The Scutellarin and 3,5-dicaffeoylquinic acid used in this study were produced by Chengdu Pusi Biotechnology Co., Ltd.; the batch numbers are 010046 and 0066-0025. The dosage of Scutellarin was determined to be 1.1505 mg/kg/day^16,20,44–49^ and the drug concentration was 0.2301 mg/mL. The dosage of 3,5-dicaffeoylquinic acid was 0.2335 mg/kg/day^19,50–52^ and the drug concentration was 0.0467 mg/mL. The volume of the above drugs was 5 mL/kg/day.

Red tetrazolium is produced by Shanghai Lingjin Fine Chemical Co., Ltd., Evans blue is produced by Shanghai Ruji Biotechnology Development Co., Ltd. and Toluidine blue (imported packaging) is produced by Shanghai Ruji Biotechnology Development Co., Ltd. The total RNA extraction reagent is produced by Invitrogen, USA. The PrimeScript RT reagent Kit and SYBR Premix Ex Taq II Kit are produced by Bao Bioengineering (Dalian) Co., Ltd.; the BCA protein concentration determination kit is from Nanjing Kaiji Biotechnology Co., Ltd.; claudin-5, iNOS, MMP-9, ZO-1, occludin, the β-actin antibody rabbit polyclonal antibody and the biotinylated goat anti-mouse IgG (H + L) antibody were purchased from British Abcam-Aibo (Shanghai) Trading Co., Ltd.

### Network pharmacology research

#### Search and screening of chemical components

According to databases such as TCMSP and TCMD@Taiwan, the relevant chemical components of *E. breviscapus* were searched, and the chemical components of *E. breviscapus* were fully supplemented through modern biotechnology databases such as CNKI, VIP database, Pubmed, and Wanfang database.

#### Drug and disease-related target analysis and screening

With the traditional Chinese medicine system pharmacology database and analysis platform (TCMSP) as the research scope, the target of the chemical components of traditional Chinese medicine is collected. For chemical components that have not been retrieved, use Swiss Target Prediction to query the target protein of the component. Search for disease targets by searching for ischemic stroke in GeneCards (http://www.genecards.org) and the comprehensive database of human genes and genotypes (OMIM, http://omim.org). Then screen out the target protein with a correlation coefficient greater than 2.

#### Construction of drug-component-target-disease network and target interaction analysis

The component-related targets of *E. breviscapus* and the ischemic stroke disease-related targets were intersected to obtain a common target and draw a Venn diagram. The relationship network was drawn by Cytoscape software. Import potential targets into String.

#### GO, KEGG enrichment analysis

Use DAVID Bioinformatics Resources6.8 database to conduct Gene oncology (GO) enrichment analysis and Kyoto Encyclopedia of Genes and Genomes (KEGG)^53–55^ signal pathway enrichment analysis of *E. breviscapus* to gain an in-depth understanding of the biological processes, molecular functions and cell composition involved.

### Quality control of 10 chemical components in *E. breviscapus* injection

*The Pharmacopoeia of the People's Republic of China* (2015 edition·Part 1) records that *E. breviscapus* mainly contains wild baicalin and total caffeic acid esters, using thin layer chromatography and high performance liquid phase Chromatographic method for content determination. However, it is found from the fingerprint of *E. breviscapus* injection that there are still many chemical components in *E. breviscapus* injection that have not been quantified and qualitative. Therefore, in order to further improve the quality control level of *E. breviscapus* injection, the HPLC method was used to simultaneously determine the content of 5-caffeoylquinic acid, chlorogenic acid, 4-caffeoylquinic acid, coffee acid, 1,3-dicaffeoylquinic acid, Scutellarin, isochlorogenic acid B, 3,5-dicaffeoylquinic acid, lamps armour, 4,5-dicaffeoylquinic acid in the *E. breviscapus* injection, as shown in the supplementary figure 1. In order to improve the quality control standards of *E. breviscapus* injection and the re-evaluation of the drug after the market, it also provides a research basis for the effectiveness evaluation of the chemical components of *E. breviscapus* injection in protecting BBB damage caused by cerebral ischemia.

The chromatographic conditions and methodology of *E. breviscapus* injection were investigated by high performance liquid chromatography. Taking the peak areas of absorption wavelengths of 10 chemical components as a reference, the best wavelength is selected as 327 nm; 0.4% phosphoric acid solution-acetonitrile is selected as the best mobile phase, the gradient elution program is shown in the supplementary table 1 and the chromatographic peaks have good resolution and symmetrical peak shapes under this mobile phase condition; Under the condition of 30 °C column temperature, the chromatographic resolution of each component is good; under the condition of 0.8 mL/min flow rate, the chromatographic resolution of each component is good; Inertsil ODS-3 column (250 × 4.6 mm, 5 μm) separation The effect is the best, which determines the best chromatographic conditions. Under these conditions, the *E. breviscapus* injection solution was sampled, and the chromatographic resolution was good, the peak shape was symmetrical, and each peak met the requirements of HPLC content determination. The chromatogram is shown in the supplementary figure 2.

HPLC method was used for quantitative analysis, and the linear relationship of 10 chemical components of *E. breviscapus* injection was investigated. The linear relationship between peak area of 10 chemical components and sample content was measured, and the precision, reproducibility, stability and addition The RSD% of sample recovery rate is less than 3%. In terms of content determination, they are all in line with the scope of *the Pharmacopeia of the People's Republic of China* (2015 edition·Part 1).

In summary, the method is stable, reliable, and suitable for the determination of multiple components of *E. breviscapus* injection, and can provide a reference for improving the quality control standards of *E. breviscapus* injection and the evaluation of the drug after it is marketed.

### Animals

Chengdu Dashuo Biotechnology Co., Ltd. provided a total of 399 male SD rats at SPF grade with their weights ranging from 320 to 350 g and bearing laboratory animal production license number SCXK(Chuan)2020-030, Laboratory Animal Quality Qualification Certificate No. 0015982, 0015985, etc. The rats were randomly divided into the following groups: sham operation, model control, positive drug (Nimodipine injection) control, *E. breviscapus* injection, Scutellarin and a 3,5-dicaffeoylquinic acid group. The group was subdivided into three subgroups for the BBB permeability test, cerebral infarction ratio observation, brain histomorphology, RT-PCR and WB studies; There are 6 groups with 3 subgroups in each group, ensuring 6 animals in each subgroup and 18 animals in each group in the final statistics. The above experimental animals were all examined and approved by the Experimental Animal Ethics Committee of Chengdu University of Traditional Chinese Medicine which agreed that the experiment was in line with animal protection, animal welfare, and ethical principles, and that it complied with the relevant regulations of the National Laboratory Animal Welfare Ethics, the number is 2020-14.

### The model of cerebral I/R injury

In this study, we use the reference method from the previous research of^30,32^ 20% urethane solution was injected intraperitoneally (0.6 mL/100 g) to anesthetize the rats. The right common carotid artery (CCA) was isolated and a suture thread was placed proximal to the end of the needle. The hemostatic forceps held the end of the thread to pull. The external carotid artery (ECA) and the internal carotid artery (ICA) continued to separate along the direction of the CCA, ligating the ECA at the bifurcation. The ligature of the ECA was clamped by a hemostatic forcep, and the pterygopalatine artery was separated inward along the ICA and ligated at the bifurcation in a similar manner. Then, a small incision was cut between the ligature at the distal end of the external carotid artery and the loose end of the proximal end. The vascular iliac line was inserted into the ICA with the help of a self-made needle. The insertion length was about 20 mm, and then surgical wounds were sterilized and disinfected. Intraoperative warming was conducted until each animal was awake. In this context, the model group means the stroke group. Reperfusion was performed two hours after the middle cerebral artery was blocked while rats in the sham operated group were prepared only at the arteries without any modeling.

Nimodipine injection, *E. breviscapus* injection, Scutellarin solution, 3,5-dicaffeoylquinic acid solution or sterilization injection water were injected twice in the course of the experiment. The first injection was given through the right common jugular vein or its branches after the model was established. The second administration was 23.5 h after cerebral ischemia by slow femoral vein injection.

### Effectiveness evaluation

#### Neuroethology evaluation

A neuroethology assessment was conducted on the three groups of rats by means of Longa and Bederson's 5-point system at various time points of cerebral ischemia namely at 2 h and 24 h^56^ (0 point: no symptoms of nerve damage; 1 point: unable to fully extend the left forepaw; 2 points: turn to the left; 3 points: dump to the left; 4 points: unable to walk, loss of consciousness)^31^.

#### Cerebral infarction rate

At 24 h after cerebral ischemia, 20% urethane solution was injected intraperitoneally (0.6 mL/100 g) to anesthetize the rats. The rats died after 10–15 mL of whole blood was collected from abdominal aorta, and the whole brain was immediately taken out for TTC staining and infarction rate calculation. TTC staining methods were as follows: brain tissue preparation: after 24 h of cerebral ischemia, the rats were observed in neurobehavioral score of cerebral ischemia, sacrificed under anesthesia, and the whole brain was removed. The water on the surface of the brain tissue was dried with a filter paper, weighed, and placed in a − 20 °C refrigerator for quick freezing for about 20 min, so as to be convenient for section. TTC preparation: 1% TTC solution was prepared with normal saline, and it was ready for use. Slice: Cut 4 knives along the coronal shape and cut into 5 pieces, each piece is about 2 mm thick. The slices were placed in phosphoric acid buffer solution of 1%TTC, covered with tin foil, and placed in a 37 temperature box for 30 min. The slices were turned over every 10 min to make uniform contact with the staining solution. The normal brain tissue is rose-red and the subcortical infarct is pale and well defined. Cerebral infarction ratio (%) = infarct fraction weight (g) 100/whole brain weight (g).

#### Brain histopathology

At 24 h after cerebral ischemia, 20% urethane solution was injected intraperitoneally (0.6 mL/100 g) to anesthetize the rats. The rats were killed with cervical dislocation. The whole brain was immediately removed and the ischemic side brain tissue was dried with filter paper. Some ischemic side brain tissue was fixed in 10% formaldehyde, then the pathological changes of the brain tissue were detected by HE staining and the expression of Nissl body was detected by toluidine blue staining. The rest of the brain tissue was fixed in 3% glutaraldehyde solution. The ultrastructural and pathological nerve cell and BBB changes in each rat brain were observed by transmission electron microscopy.

A microscopic camera system was used to collect images of slices, observe all of the tissues and gross lesions, select the areas to be observed to collect the images and observe specific lesions. We found: − indicates that no lesions were found. + indicates that small focal grey and white matter edema, cell lysis; small focal grey and white matter cell atrophy, cytoplasm and nuclear hyperchromatism; focal small cell proliferation (mainly glial cells); mild perivascular edema; mild vascular congestion, hemorrhage; a little inflammatory cell infiltration; mild atrophy of gray matter thin; and a little ependymal cell proliferation, in line with one of them. There was also: ++ indicates that multifocal grey and white matter edema, cell lysis; multifocal grey and white matter cell atrophy, hyperchromatic cytoplasm and nucleus; focal multicellular proliferation (mainly glial cells); moderate vascular congestion and hemorrhage; and more inflammatory cell infiltration. Moderate perivascular edema was found in one case. In addition, we found +++ indicates that diffuse grey and white matter edema, cell lysis; multifocal grey and white matter cell atrophy, hyperchromatic cytoplasm and nuclei; focal massive cell proliferation (mainly glial cells); severe vascular congestion and hemorrhage; and a large number of inflammatory cell infiltrations, in line with one of them.

#### Number of Nissl bodies in brain tissue

The thickness of Nissl staining slices was 1–5 μm. We next performed anti-stripping treatment of glass slides, soaking with APES, putting the slides in an oven at 60 °C for 60 min to make the slices adhere tightly; routine dewaxing of slices to water; putting into a 1% toluidine blue water solution preheated to 50 °C, dyeing at 56 °C for 20 min; washing with distilled water; soaking 70% alcohol for 1 min; differentiating 95% alcohol under a microscope that showed clear Nissl body and quick anhydrous alcohol. Dehydration, xylene transparent, neutral gum sealing and finally, image acquisition of slices was carried out using a microscopic camera system. All of the tissues and gross lesions were observed in each slice, and then the areas to be observed were selected in order to collect images for the observation of the specific lesions. The detection was observed and counted under a light microscope. Each brain tissue was selected to collect 400-fold images from three visual fields (i.e., different regions of the hippocampus, respectively). The mean value of each visual field was taken as the cell density of the sample after counting.

#### Measurement of blood–brain barrier permeability (Evans Blue method)

At 24 h, a 2% EB solution (4 mL/kg) with normal saline was injected slowly. The eyes, limbs and caudal veins of the rats rapidly turned blue. After two hours, the right cerebral hemisphere (i.e., the ischemic brain tissue) was weighed by heart perfusion. The supernatant was incubated in formamide solution, at 60 °C for 24 h, and the supernatant was retained. The OD value of the supernatant was determined by an ultraviolet spectrophotometer (lambda = 620 nm). Amine solution was used as the sham operation.

**Figure 2:**
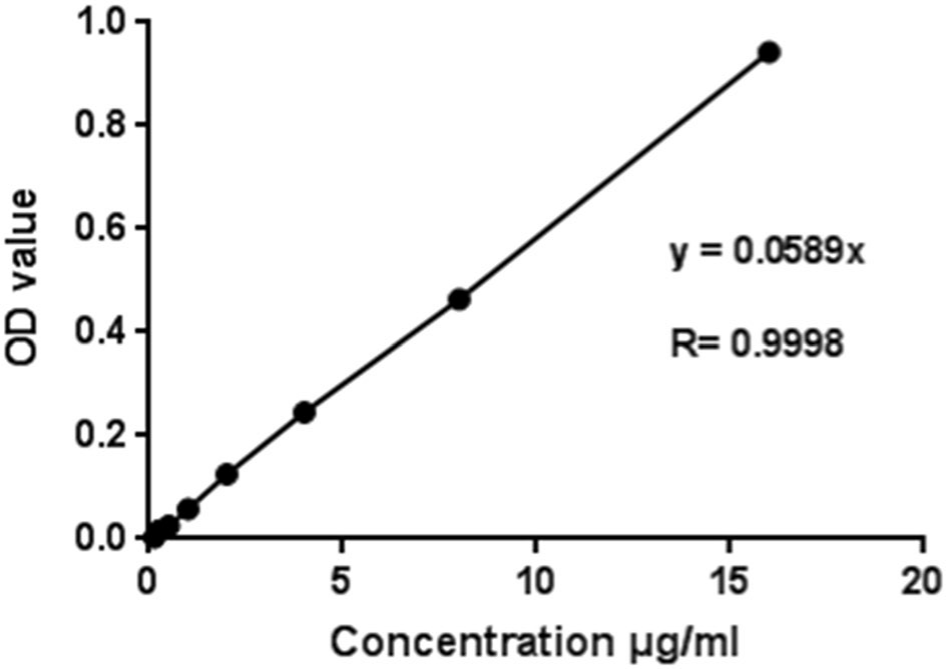
Evans Blue standard curves.

Different OD values were measured with different gradient concentrations of formamide in the EB solution and standard curves were drawn. The concentration of the standard curve of the EB was 16 μg/mL, 8 μg/mL, 4 μg/mL, 2 μg/mL, 1 μg/mL, 0.5 μg/mL, 0.25 μg/mL, 0.125 μg/mL, 0.0625 μg/mL and 0.03125 μg/mL, respectively (Fig. 2).

According to the standard curve of the EB, the content of the EB in the sample to be measured was calculated as follows: EB content in brain tissue (μg/g) = EB content (μg/mL) * formamide capacity (mL)/brain tissue weight (g) in the samples to be measured^56–68^.

#### Ultrastructural observation of the blood–brain barrier (transmission electron microscope)

The ischemic brain tissue was pre-fixed with 3% glutaraldehyde and dehydrated step by step with acetone. The dewatered tissues were then successively treated with a dehydrating agent and epoxy resin (Epon 812) osmotic solution in the proportions of 3:1, 1:1 and 1:3 at 30–60 min for each step. The infiltrated sample block was placed in the appropriate mold, and the embedding solution poured into the mold to embed the solid matrix (embedding block) formed by heating and polymerization for the next section. Ultra-thin slices about 50 nm thick were prepared by an ultra-thin slicing machine, floated on the liquid surface of the knife trough, then fished to the copper mesh, dyed and observed by electron microscopy. Double staining (i.e., uranium acetate and lead citrate staining) was conducted for 15–20 min at room temperature. We then observed the ultrastructural changes of neurons and BBBs (including the vascular endothelial cells, TJs between the endothelial cells and the basement membranes) in each cerebral cortex and photographed them under a Hitachi H-600IV transmission electron microscope.

### Study on the mechanism

#### Real-time PCR

The expression of iNOS, MMP-9, claudin-5, occludin and ZO-1 mRNA in the brain tissue of the rats was analyzed by RT-PCR after 24 h cerebral ischemia reperfusion. Total RNA extraction uses the total RNA kit extraction method as required by the manufacturer. The removal reaction and reverse transcription reaction of the genomic DNA were carried out in turn. After polymerase chain reaction amplification, the expression of the mRNA was analyzed by the real-time fluorescence quantitative PCR method, and the β-actin gene was used as a reference for the standardization of different transcription values. The data analysis is based on the formula F = 2^−△△CT^, △△CT = (the average CT value of genes in drug group-average CT value of internal reference gene in drug group) − (average CT value of gene in blank group-average CT value of internal reference gene in blank group). The relative expression of iNOS, MMP-9, claudin-5, occludin and ZO-1 mRNA was calculated, and the primer and base sequence were used in this study (Table 1).

**Table 1:** The primers and base sequences used in this test.

Primer name	Upstream	Downstream
β-Actin	gaagatcaagatcattgctcct	tactcctgcttgctgatcca
iNOS	cggaagagacgcacaggcagaggtt	gaaggcagcaggcacacgcaatgat
MMP-9	ccaccaccgccaactatgaccaggat	gtactgcttgcccaggaagacgaagg
ZO-1	ggagtgtcattgccgtcgcatgtaga	tgtatggtggctgctcaaggtctctg
Claudin-5	tcgtcatcgtgatgtgcatcgctgta	ccgtaaccgtagccgtaaccgtaacc
Occludin	tctgctggttcgccaacatcgtagtc	cgtggttcgccttggtgctgagta

#### Western blot

A sample of brain tissue was frozen in the rats with liquid nitrogen, thawed in a 37 °C water bath and then transferred to a 2 mL EP tube. We then added RIPA lysate to each tube (according to rat brain tissue: lysate = 1:10), cracking 10 min on ice, collecting lysate at 4 °C, 12,000 r/min centrifugal for 15 min. In the collection of supernatant, the protein concentration was determined using the BCA protein quantitative kit. Sample determination, protein denaturation, sample electrophoresis, transfer membrane, closure, incubation of antibodies (one resistance concentration: claudin-5 1:500; INOS 1:200; MMP-2 1:1000; MMP-9 1:200; occludin 1:5000; ZO-1 1:1000; and β-actin 1:5000), development and fixing, image analysis with a gel image analysis imaging system for scanning analysis was conducted. The results are revealed in the relative expression of the target protein (i.e., the integral light density value /internal ginseng integral light density value of the target protein).

### Statistical analysis

SPSS21.0 software was used for our statistical analysis. The measurement data were expressed as mean ± standard deviation ($\overline{x} \pm s$). One-way ANOVA was used for the inter-group difference test, the two-tailed test will be used to compare sham operation group, positive drug (Nimodipine injection) control group, *E. breviscapus* injection group, Scutellarin group and a 3,5-dicaffeoylquinic acid group with model group. A nonparametric test was used to determine the rats’ neurological function scores. There were six animals in each group.

### Consent for publication

Written informed consent for publication was obtained from all participants.

### Ethics approval and consent to participate

Our animal experiments were approved by Chengdu Dashuo Biotechnology Co., Ltd. and bearing laboratory animal production license number SCXK(Chuan)2020-030, Laboratory Animal Quality Qualification Certificate No. 0015982, 0015985, etc., and conformed to the guide for the Care and Use of Laboratory (approve number SYXK(Chuan)2020-124). The above experimental animals were all examined and approved by the Experimental Animal Ethics Committee of Chengdu University of Traditional Chinese Medicine which agreed that the experiment was in line with animal protection, animal welfare, and ethical principles, and that it complied with the relevant regulations of the National Laboratory Animal Welfare Ethics, the number is 2020-14.

### Statement

All methods are implemented in accordance with the relevant guidelines and regulations. All content of the article follows the ARRIVE guidelines.

## Results

### Network phrmacology rsearch

#### Network analysis of “*E. breviscapus*-chemical components-drug disease target-ischemic stroke”

The active ingredients of “OB > 30%” and “DL > 0.18%” were screened according to the ADME information of Chinese medicine ingredients. Obtained the chemical components of 11 *E. breviscapus * (supplementary table 2), and searched for “ischemic stroke” in GeneCards (http://www.genecards.org), the comprehensive database of human genes and genotypes (OMIM, http://omim.org) to query disease targets, and screen out target proteins with a correlation coefficient greater than 2. Enter the obtained target of ischemic stroke and the target of *E. breviscapus* into the UniProt database (http://www.uniprot.org) to search, and obtain the Gene Symbol of all the targets for subsequent analysis. Use Venny2.1 (http://bioinfogp.cnb.csic.es/tools/venny/). Through the search, 6947 ischemic stroke disease targets were obtained. After matching with the potential targets of *E. breviscapus* drug action, a total of 91 relevant targets of *E. breviscapus* for the treatment of ischemic stroke diseases were obtained (supplementary table 3), and the Venn diagram was drawn (supplementary figure 3a). And get the intersection target.

Using Cytoscape 3.2.1 software, the effective chemical components of *E. breviscapus* and the corresponding targets for the treatment of ischemic stroke were assembled to construct a notework diagram of “chemical components-targets-ischemic stroke”(supplementary figure 3b). The network includes 104 nodes and 554 edges. And through different colors and shapes to visualize it, in order to more intuitively and clearly show the relationship between the chemical composition of *E. breviscapus* and the target of ischemic stroke disease. In the figure, the red diamond represents *E. breviscapus*; the yellow triangle represents the active chemical components in *E. breviscapus*; the blue hexagon represents ischemic stroke disease; the green origin represents the target point of the joint action of drugs and diseases.

#### “Protein–protein” network topology analysis

Use the string plug-in to analyze the network topology to obtain the network topology parameters of the target gene (supplementary figure 3c). Through the topology analysis, we can get the relationship of the target gene interaction. Screen 18 important target genes, and get the target gene interaction diagram. Among them, the most interacting genes such as PTGS1 and PPARG are important related genes in the ROS/RNS pathway of *E. breviscapus* treatment of cerebral ischemia. It confirms the protective effect of *E. breviscapus* on the related pathways in the treatment of cerebral ischemic stroke.

#### GO enrichment analysis of *E. breviscapus*

The DAVID database was used to perform GO enrichment analysis on the *E. breviscapus* target based on the terms of biological process (BP) and molecular function (MF) (supplementary figure 3d). The biological process and molecular function of *E. breviscapus* were analyzed, and the first 20 processes with the least significance were selected. Through GO analysis of protein binding process, apoptosis process, enzyme binding process, positive regulation of gene expression, steroid hormone receptor activity, negative regulation of apoptosis process, cell response to hydrogen peroxide, etc. that may be related to ischemic stroke.

#### KEGG enrichment results

KEGG analysis was performed on 91 potential targets, and a total of 87 signal pathways were obtained. There are 15 signal pathways related to stroke, as shown in the supplementary figure 3e. Among them, the HIF-1 signaling pathway (hsa04066), VEGF signaling pathway (hsa04370) related to angiogenesis; PI3K-Akt signaling pathway (hsa04151), Wnt signaling pathway (hsa04310), FoxO signaling pathway (hsa04068), MAPK signaling pathway (hsa04010), AMPK signaling pathway (hsa04152), ErbB signaling pathway (hsa04012), RAS signaling pathway (hsa04014) and Apoptosis (hsa04210) are related to cell cycle; Sphingolipid signaling pathway related to cell signaling (hsa04071); Neural-related neurotrophin signaling pathway (hsa04772); Lipid-related cAMP signaling pathway (hsa04024); Focal adhesion (hsa04510) related to protein transduction; NF-kappa B signaling pathway (hsa04064) related to inflammation.

The study found that 44 of 91 potential targets are related to these signaling pathways. The related chemical components include luteolin, kaempferol, quercetin, 1-hydroxy-2,3,5-trimethoxy-xanthone, baicalein, etc., which may be the potential active ingredient in the treatment of ischemic stroke. According to information and research investigations, it is found that PI3K-Akt signaling pathway is closely related to nerve damage, as shown in the supplementary figure 3f, the KEGG copyright permission has been obtained from Kanehisa Laboratories. There are 17 potential targets related to it. Among them, CCND1, EGFR, RELA, VEGFA, and CASP9 have more freedom. The mechanism of breviscapine in the treatment of ischemic stroke is related to neuroprotection, and the results of network pharmacology need further experimental verification.

### Protective effect of brain tissue

#### Neurobehavioral grading

The rats in our stroke group, compared with the sham-operated group, showed obvious neurobehavioral abnormalities at 2 h and 24 h after cerebral ischemia (*P* < 0.01), which were manifested as left turn, left tilt, left forefoot unnaturally straightening, etc. Compared with the stroke group, the Nimodipine injection, *E. breviscapus* injection, Scutellarin and 3,5-dicaffeoylquinic acid groups at 2 h after cerebral ischemia. All of the rats showed neurobehavioral abnormalities (*P* > 0.05). At 24 h after cerebral ischemia, the neurobehavioral abnormalities of rats in the Nimodipine injection, *E. breviscapus* injection, Scutellarin and 3,5-dicaffeoylquinic acid groups all recovered. The neurobehavioral recovery of rats in the Nimodipine injection, *E. breviscapus* injection and Scutellarin groups was significant (*P* < 0.01 or *P* < 0.05) (Table 2).

**Table 2:** Neurobehaviology of rats.

Group	Dose	n	Neurobehavioral grading in cerebral ischemia 24 h					
			−	+	++	+++	++++	*P* value
Sham operation group	–	6	6	0	0	0	0	0.002**
Model group	–	6	0	2	3	1	0	1
Nimodipine injection group	0.5 mg/kg	6	5	1	0	0	0	0.036*
*E. breviscapus* injection group	5 mL/kg/day	6	5	1	0	0	0	0.036*
Scutellarin group	1.1505 mg/kg	6	6	0	0	0	0	0.002**
3,5-Dicaffeoyl quinic acid group	0.2335 mg/kg	6	4	2	0	0	0	0.094

*Compared with the model group, *P < 0.05 and **P < 0.01.

#### *E. breviscapus* treatment reduces infarct size

**Figure 3:**
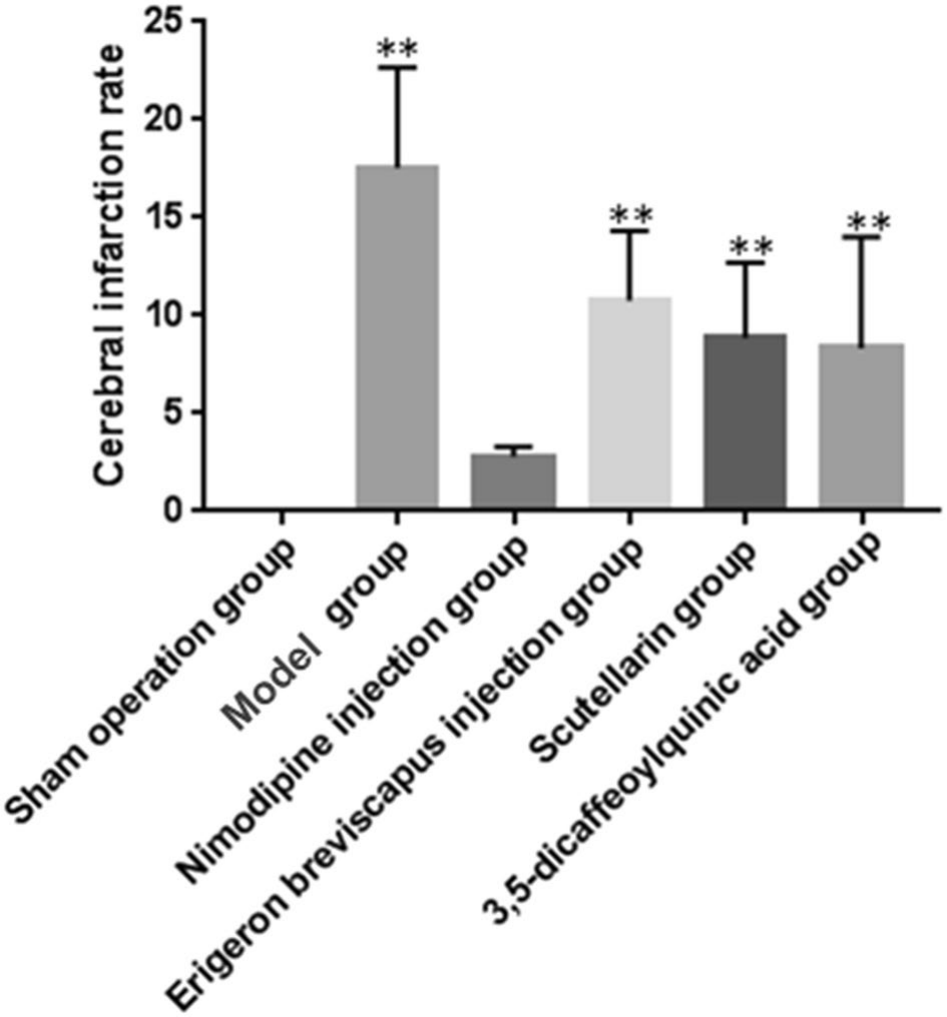
Changes of the cerebral infarction rate in rats. The values are presented as mean ± SD. (n = 6), * compared with the model group, *P < 0.05 and **P < 0.01.

**Figure 4:**
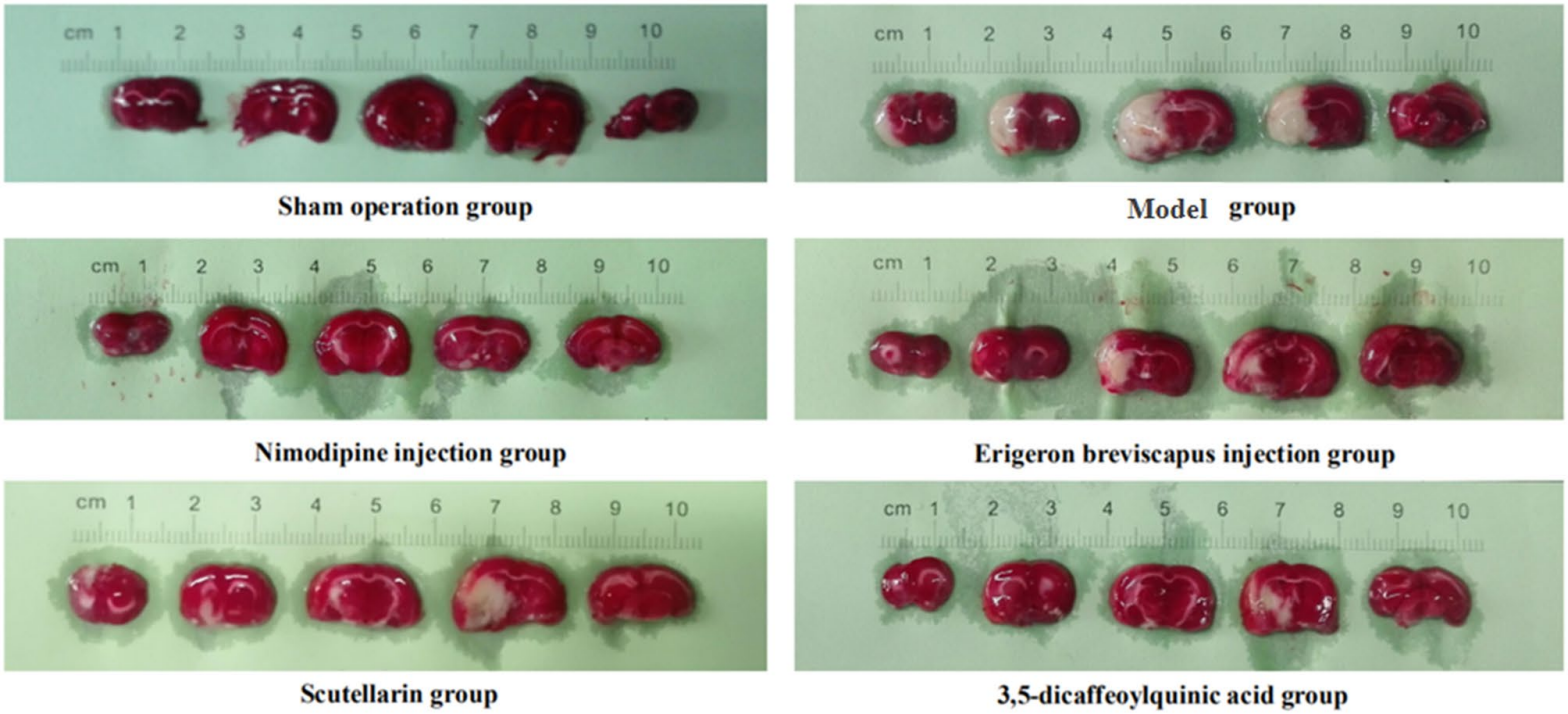
*E. breviscapus* treatment reduces infarct size in rats: ① the pale region of the midbrain is the cerebral infarction area; and ② selected the brain slices of one typical animal in each group.

The cerebral infarction rate of the model group, compared with the sham-operated group, increased significantly at 24 h of cerebral ischemia (*P* < 0.01). And, compared with the model group, the cerebral infarction rate of the Nimodipine injection, *E. breviscapus* injection, Scutellarin and the 3,5-dicaffeoylquinic acid groups decreased significantly at 24 h of cerebral ischemia (*P* < 0.01), as shown in the Figs. 3 and 4.

#### Histopathological ischemic brain changes

**Figure 5:**
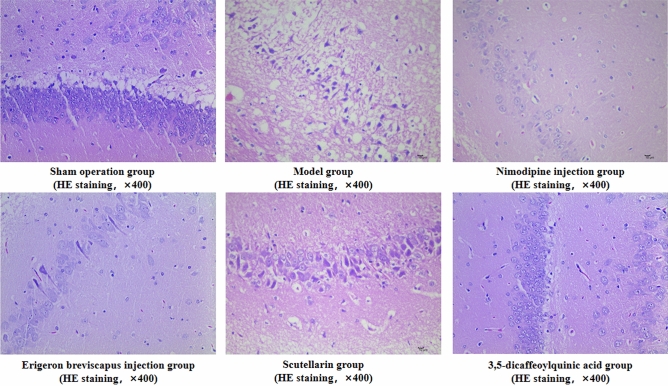
Histopathological changes of ischemic brain in rats: ① in each group, 400-fold images were collected from one area according to tissue size and expression; and ② the arrow indicates that 1 is normal vertebral cells and 2 is degenerated vertebral cells (Scale bar: 10 μm.)

Compared with the sham operation group, there was edema in the gray and white matter of the ischemic lateral brain tissue in our model group, diffuse edema of gray and white matter in serious injury, massive cell dissolution necrosis, proliferation of small glial cells and small necrosis of vertebral cells (*P* < 0.01). And, compared with the model group, the number of gray matter and white matter edema in the ischemic lateral brain tissue of the Nimodipine injection, *E. breviscapus* injection, wild Baicalin and 3,5-two coffee-quinine acid groups was less, and the degree of edema decreased. In addition, the number of dissolved necrosis of cells, the number of small glial cell hyperplasia and the number of vertebral cell necrosis were reduced (*P* < 0.01) (Table 3; Fig. 5).

**Table 3:** Histopathological ischemic brain changes in rats.

Group	Dose	n	Histopathological grading of ischemic brain in rats					
			−	+	++	+++	++++	*P *value
Sham operation group	–	6	6	0	0	0	0	0.002**
Model group	–	6	0	0	4	2	0	1.000
Nimodipine injection group	0.5 mg/kg	6	3	2	0	1	0	0.031*
*E. breviscapus* injection group	5 mL/kg/d	6	1	4	0	1	0	0.030*
Scutellarin group	1.1505 mg/kg	6	2	1	3	0	0	0.031*
3,5-Dicaffeoylquinic acid group	0.2335 mg/kg	6	3	1	2	0	0	0.016*

*Compared with the model group, *P < 0.05 and **P < 0.01.

#### Number of Nissl bodies in brain tissue

**Figure 6:**
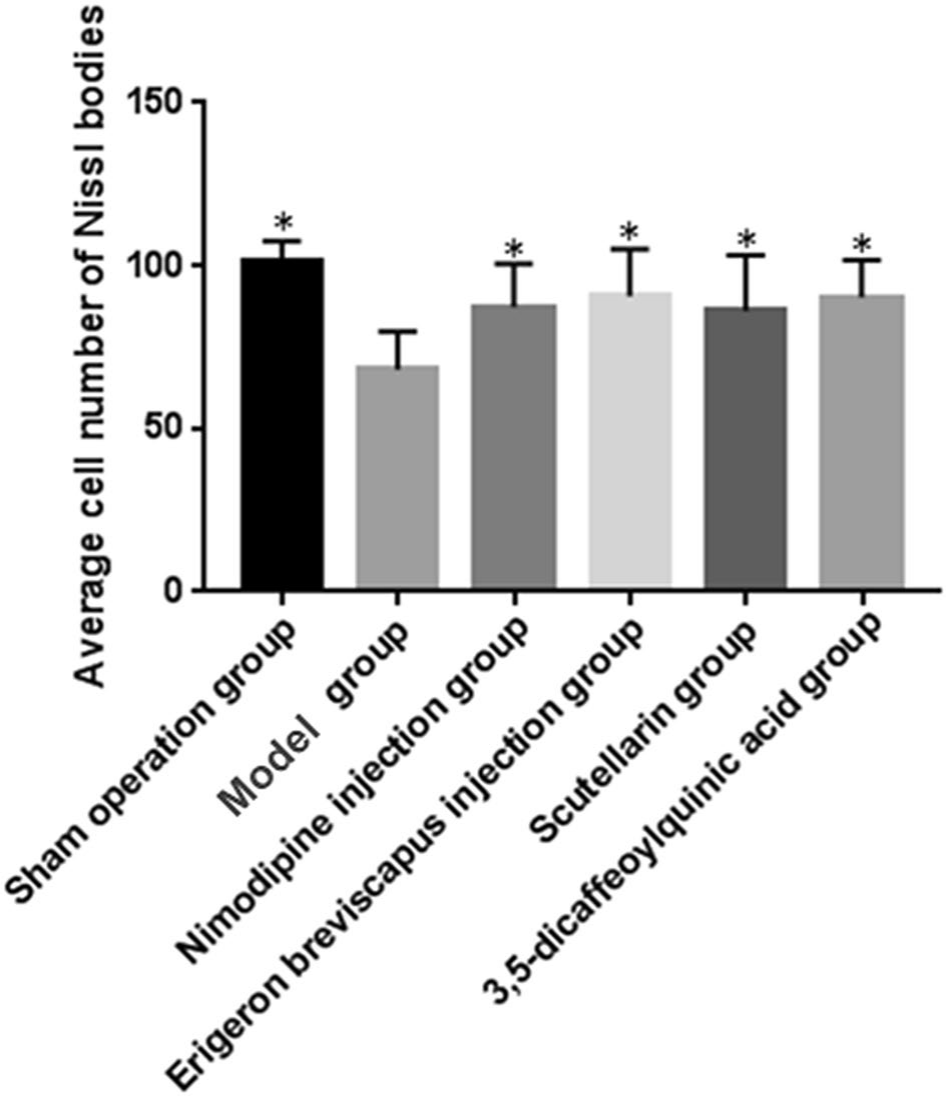
Average number of Nissl somatic cells in the ischemic brain tissues of rats. The values are presented as mean ± SD. (n = 6), * compared with the model group, *P < 0.05 and **P < 0.01.

**Figure 7:**
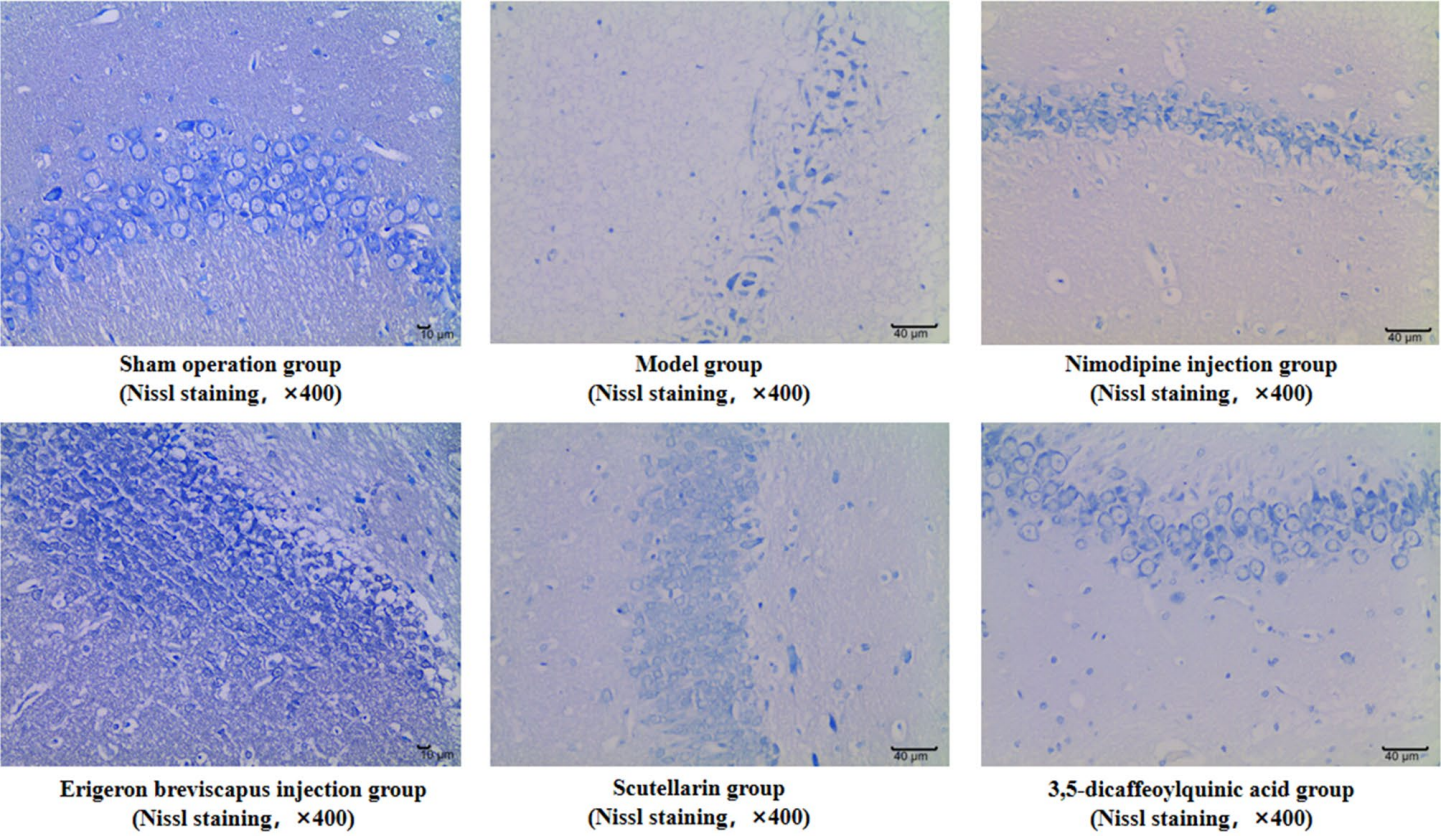
Nissl bodies in the ischemic brain tissue of rats: ① three visual fields were selected for each brain tissue to collect 400-fold images, and the mean value of each visual field was taken as the cell density of the sample after counting; ② the arrow indicates Nissl body. (Scale bar: Sham operation group − 10 μm; Model control group − 40 μm; Nimodipine injection group − 40 μm; *E. breviscapus* injection group − 10 μm; Scutellarin group − 40 μm; 3,5-dicaffeoylquinic group − 40 μm.)

The average number of Nissl bodies in the ischemic brain tissue of rats in our stroke group, compared with the sham-operated group, decreased significantly (*P* < 0.01). And, compared with the stroke group, the average number of Nissl bodies in the ischemic brain tissue of rats in the Nimodipine injection, *E. breviscapus* injection, Scutellarin and 3,5-dicaffeoylquinic acid groups increased significantly (*P* < 0.01), as shown in the Table 4, Figs. 6 and 7.

**Table 4:** Number of Nissl bodies in brain tissue and BBB permeability.

Group	Dose	n	Number of Nissl bodies in brain tissue		BBB permeability (EB method)	
			$\overline{x} \pm s$	*P *value	$\overline{x} \pm s$	*P* value
Sham operation group	–	6	101.33 ± 6.06	0.000**	3.526 ± 0.376	0.000**
Model group	–	6	68.17 ± 11.48	–	12.964 ± 4.844	–
Nimodipine injection group	0.5 mg/kg	6	87.33 ± 13.29	0.015*	4.214 ± 0.871	0.000**
*E. breviscapus* injection group	5 mL/kg/day	6	90.50 ± 14.53	0.005**	7.205 ± 1.332	0.001**
Scutellarin group	1.1505 mg/kg	6	86.00 ± 17.22	0.022*	7.035 ± 1.595	0.001**
3,5-dicaffeoylquinic acid group	0.2335 mg/kg	6	90.17 ± 11.58	0.006**	8.107 ± 1.695	0.005**

### Permeability of the BBB in brain tissue

#### BBB permeability (EB method)

**Figure 8:**
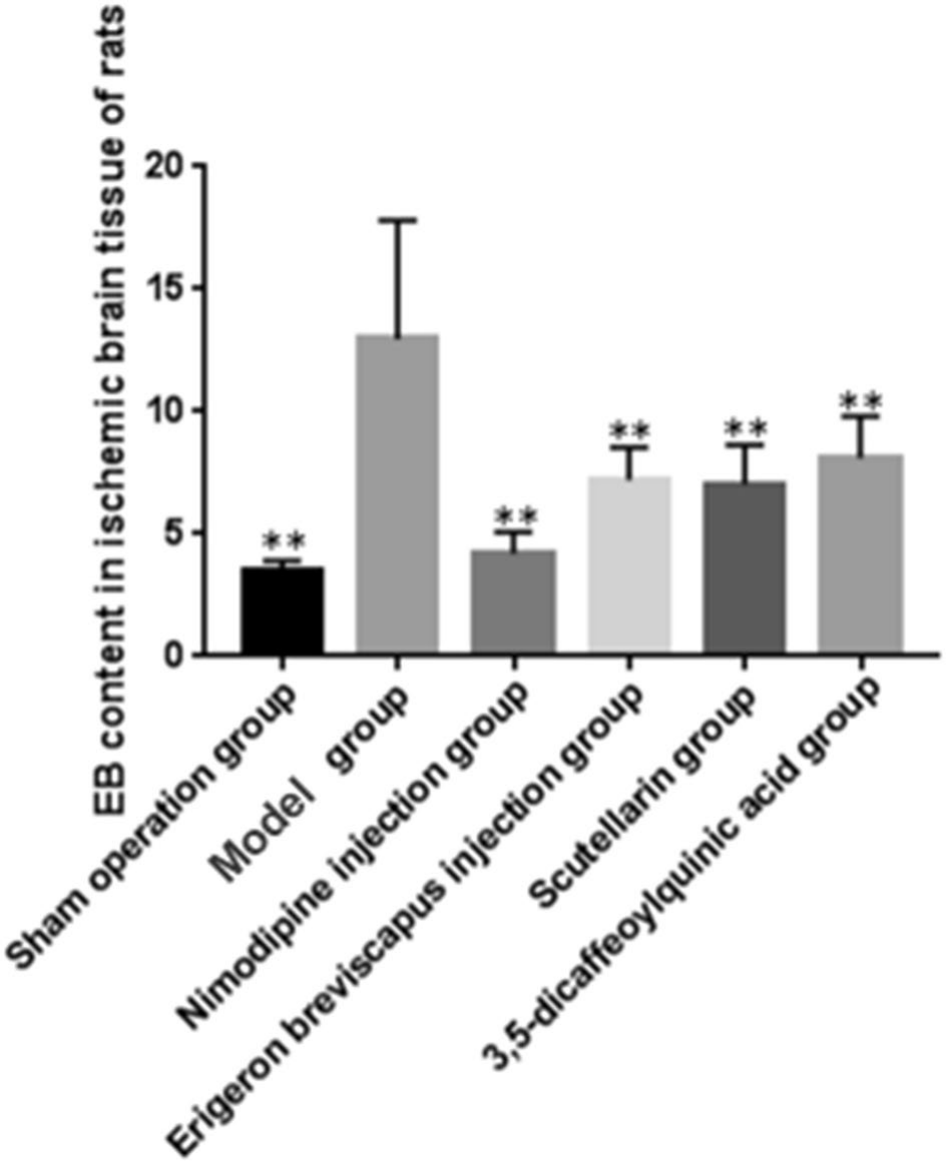
BBB permeability in the ischemic brain tissue of rats. The values are presented as mean ± SD. (n = 6), * compared with the model group, *P < 0.05 and **P < 0.01.

**Figure 9:**
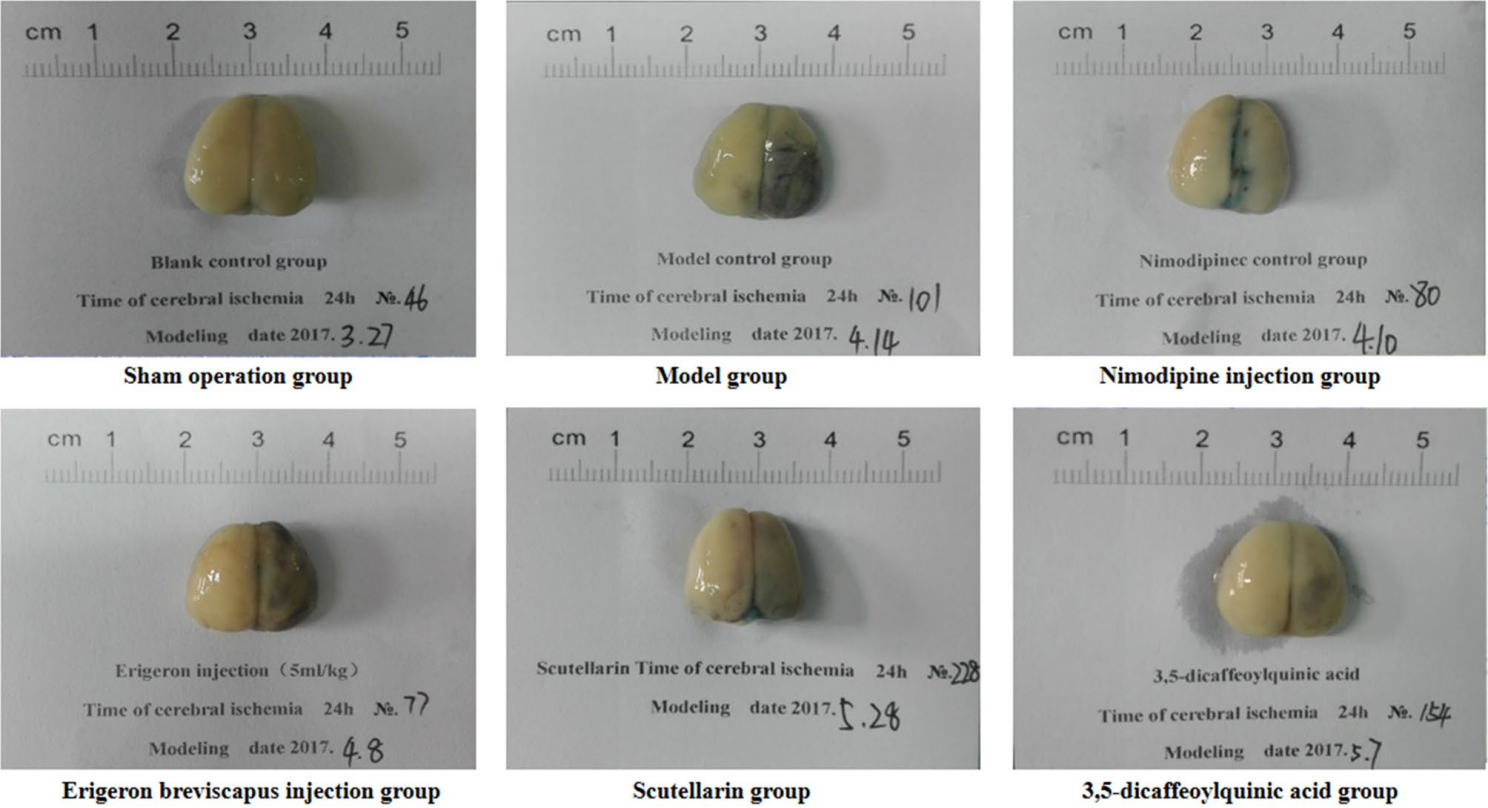
BBB permeability in the ischemic brain tissue of rats (EB staining). The blue area in the picture is dyed with EB.

The content of EB in the ischemic brain tissue of our model group, compared with sham operation group, increased significantly (*P* < 0.01). And, compared with model group, the content of EB in the ischemic brain tissue of the Nimodipine injection, *E. breviscapus* injection, Scutellarin and 3,5-dicaffeoylquinic acid groups decreased significantly (*P* < 0.01), as shown in the Table 4, Figs. 8 and 9.

#### Ultrastructure of neurons (transmission electron microscopy)

In the sham operation group, the ultrastructure of neurons was normal, the nucleus of neurons was round and the membrane structure was complete. In addition, the chromatin was evenly distributed and the structure of mitochondria, rough endoplasmic reticulum and ribosome in the cytoplasm was clear.

The nuclear membrane structure of neurons in our model group, compared with the sham operation group, was incoherent and the distribution of chromatin was uneven. Mitochondria, rough endoplasmic reticulum, ribosome and other organelles in the cytoplasm were partially dissolved and disappeared, and the structure was not clear.

**Figure 10:**
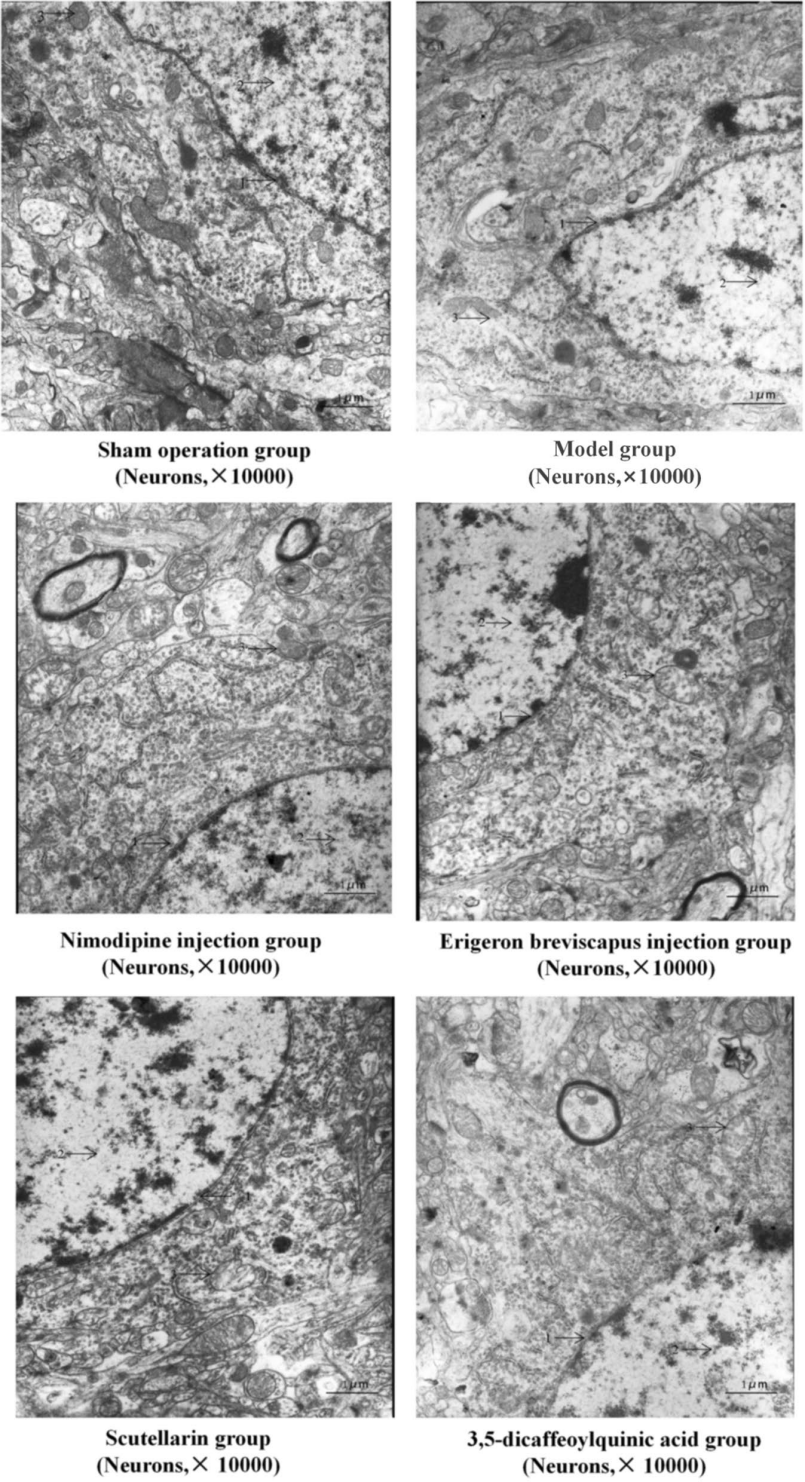
Ultrastructure of neurons in the ischemic brain tissue of rats: 1. nuclear membrane; 2. nuclear chromatin; and 3. mitochondria and other organelles in the cytoplasm.

The nucleus of neurons in the Nimodipine injection group, compared with our model group, was round and the chromatin in the nucleus was uniformly distributed. In addition, the structure of mitochondria, rough endoplasmic reticulum and ribosome in the cytoplasm was clear. The *E. breviscapus* injection, Scutellarin and 3,5-dicaffeoylquinic acid groups showed a round nuclear membrane structure and uniform distribution of chromatin. A few rats in the *E. breviscapus* injection, Scutellarin and 3,5-dicaffeoylquinic acid groups showed slight swelling of neurons and intracytoplasmic mitochondria, but the structure of the rough endoplasmic reticulum and ribosome was clear, and the 3,5-dicaffeoylquinic acid group showed a slight swelling of the neurons and intracytoplasmic mitochondria. The effect of the quinic acid group was better than that of the *E. breviscapus* injection and Scutellarin groups. The morphological structure of the *E. breviscapus* injection, Scutellarin and 3,5-dicaffeoylquinic acid groups was between the sham operation group and our model group (Fig. 10).

#### Ultrastructure of BBB (transmission electron microscopy)

In the sham operation group, the cerebral vascular structure was normal, the TJ between the vascular endothelial cells was not broken, the basal layer was in close contact with the endothelial cells, the glial processes were close to the basal layer and the perivascular cells were neither necrotic nor vacuolar.

Compared with the sham operation group, vascular endothelial cell necrosis was observed, the basement membrane structure was unclear, there was perivascular tissue necrosis and the perivascular glial cells dissolved and disappeared, showing vacuolar changes in the model group.

**Figure 11:**
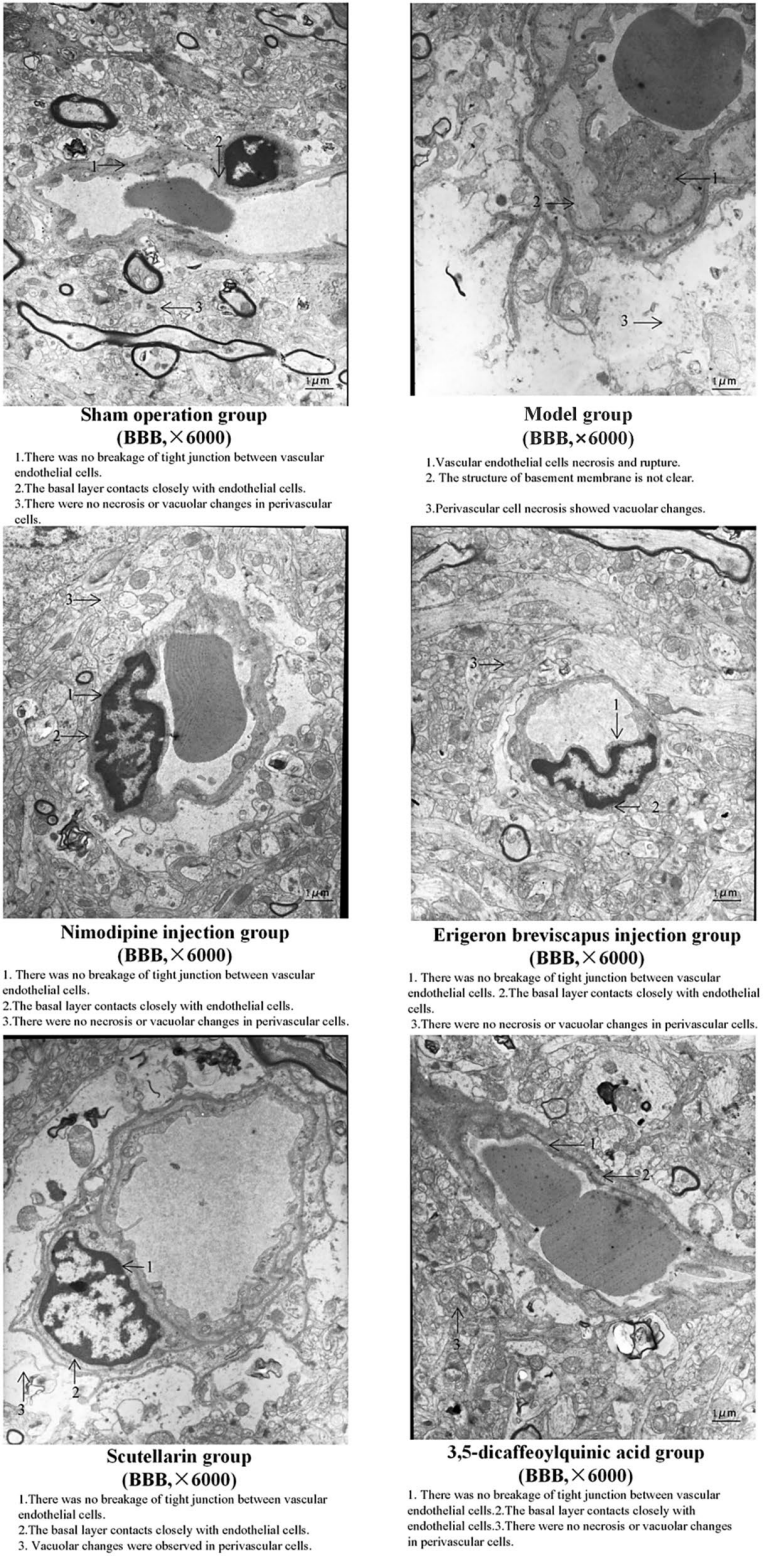
Ultrastructure of the BBB in the ischemic brain tissue of rats.

The ultrastructure of the BBB in the Nimodipine injection group, compared with our model group, was normal; the TJ between the vascular endothelial cells was not broken, the basal layer was in close contact with the endothelial cells and the glial cell processes were close to the basal layer. In addition, the *E. breviscapus* injection, Scutellarin and the 3,5-dicaffeoylquinic acid groups were improved. The cerebrovascular structure was normal in the rats studied. Only a few rats, those without vascular endothelial cell necrosis, had nuclear chromatin aggregation or cytoplasmic mitochondrial swelling, but the basal layer was in close contact with the endothelial cells, the glial processes were close to the basal layer and the morphological structure was between the prosthetic hands. Between the operation group and our model group, the effect of the *E. breviscapus* injection and the 3,5-dicaffeoylquinic acid groups was stronger than that of the Scutellarin group (Fig. 11).

### mRNA expression of the ROS/RNS-MMPs-TJs pathway in brain tissue

#### Expression of iNOS mRNA

**Figure 12:**
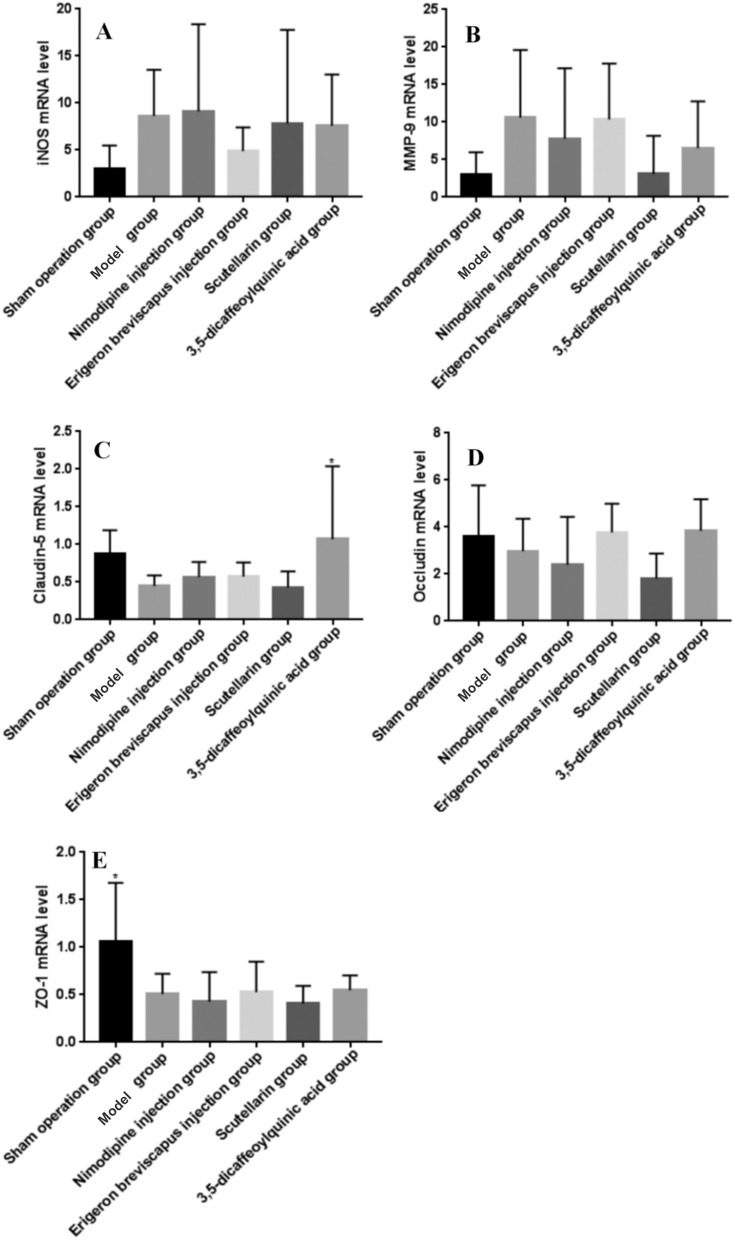
Effects of drugs on the expression of iNOS, MMP-9, claudin-5, occludin and ZO-1mRNA levels in the ischemic brain tissue of MCAO rats. (**A**–**D**) represents mRNA level analysis of expression iNOS, MMP-9, claudin-5, occludin and ZO-1. The values are presented as mean ± SD, (n = 6), * compared with the model group, *P < 0.05 and **P < 0.01.

The expression of iNOS mRNA in the ischemic brain tissue of our model group, compared with the sham operation group, tended to increase (*P* > 0.05). And, compared with the model group, the expression of iNOS in the ischemic side of the brain tissue of rats in the Nimodipine injection group showed an upward trend (*P* > 0.05), while the expression of iNOS in the ischemic side of the brain tissue of rats in the *E. breviscapus* injection, Scutellarin and the 3,5-dicaffeoylquinic acid groups showed a downward trend (*P* > 0.05) (Table 5; Fig. 12).

**Table 5:** Effects of drugs on the expression of iNOS, MMP-9, claudin-5, occludin and ZO-1mRNA levels in the ischemic brain tissue of MCAO rats.

Group	Dose	n	iNOS		MMP-9		Claudin-5		Occludin		ZO-1	
			$\overline{x} \pm s$	*P* value	$\overline{x} \pm s$	*P* value	$\overline{x} \pm s$	*P* value	$\overline{x} \pm s$	*P* value	$\overline{x} \pm s$	*P* value
Sham operation group	–	6	2.96 ± 2.53	0.140	3.05 ± 2.92	0.074	0.88 ± 0.31	0.107	3.59 ± 2.91	0.513	1.07 ± 0.61	0.007**
Model group	–	6	8.62 ± 4.92	–	10.59 ± 9.01	–	0.45 ± 0.14	–	2.98 ± 1.37	–	0.51 ± 0.21	–
Nimodipine injection group	0.5 mg/kg	6	9.12 ± 9.26	0.894	7.77 ± 9.40	0.494	0.57 ± 0.20	0.656	2.39 ± 2.04	0.528	0.43 ± 0.31	0.683
*E. breviscapus* injection group	5 mL/kg/day	6	4.88 ± 2.52	0.325	10.35 ± 7.46	0.953	0.57 ± 0.19	0.638	3.77 ± 1.22	0.396	0.53 ± 0.32	0.905
Scutellarin group	1.1505 mg/kg	6	7.84 ± 9.93	0.836	3.06 ± 5.09	0.074	0.43 ± 0.21	0.933	1.82 ± 1.06	0.217	0.41 ± 0.18	0.621
3,5-Dicaffeoylquinic acid group	0.2335 mg/kg	6	7.60 ± 5.45	0.787	6.53 ± 6.22	0.326	1.07 ± 0.97	0.022*	3.84 ± 1.34	0.354	0.55 ± 0.15	0.825

#### Expression of MMP-9 mRNA

The expression of MMP-9 in the ischemic brain tissue of our model group, compared with the sham operation group, tended to increase (*P* > 0.05). And, compared with the model group, the expression of MMP-9 in the Nimodipine injection, *E. breviscapus* injection, Scutellarin and the 3,5-dicaffeoylquinic acid groups showed a downward trend (*P* > 0.05) (Table 5; Fig. 12).

#### Expression of claudin-5 mRNA

The expression of claudin-5 in the ischemic brain tissue of our model group, compared with the sham operation group, tended to decrease (*P* > 0.05). And, compared with the model group, the expression of claudin-5 RNA in the ischemic side of rats in the Nimodipine injection and *E. breviscapus* injection groups tended to be up-regulated (*P* > 0.05), while the expression of claudin-5 in the ischemic side of rats in the 3,5-dicaffeoylquinic acid group showed a significant difference (*P* < 0.05), while the expression of claudin-5 in the ischemic side of rats in the Scutellarin group was down-regulated (*P* < 0.05); trend of adjustment (*P* > 0.05) (Table 5; Fig. 12).

#### Expression of occludin mRNA

Occludin mRNA expression in the ischemic brain tissue of our model group, compared with sham operation group, was down-regulated (*P* > 0.05). And, compared with model group, occludin gene expression in the ischemic lateral brain of rats in the *E. breviscapus* injection and 3,5-dicaffeoylquinic acid groups tended to increase (*P* > 0.05), while occludin gene expression in the ischemic lateral brain of rats in the Nimodipine injection and Scutellarin groups tended to decrease (*P* > 0.05) (Table 5; Fig. 12).

#### Expression of ZO-1 mRNA

The expression of ZO-1 in the ischemic brain tissue of our model group, compared with sham operation group, was significantly decreased (*P* < 0.05). And, compared with the model group, the expression of ZO-1 in the ischemic side of rats in the Nimodipine injection, *E. breviscapus* injection and 3,5-dicaffeoylquinic acid groups showed an upward trend (*P* > 0.05), while the expression of occludin in the ischemic side of rats in the Nimodipine injection and Scutellarin groups showed a downward trend (*P* > 0.05) (Table 5; Fig. 12).

#### Expression of the ROS/RNS-MMPs-TJs pathway related proteins in brain tissues

##### Expression of iNOS protein

**Figure 13:**
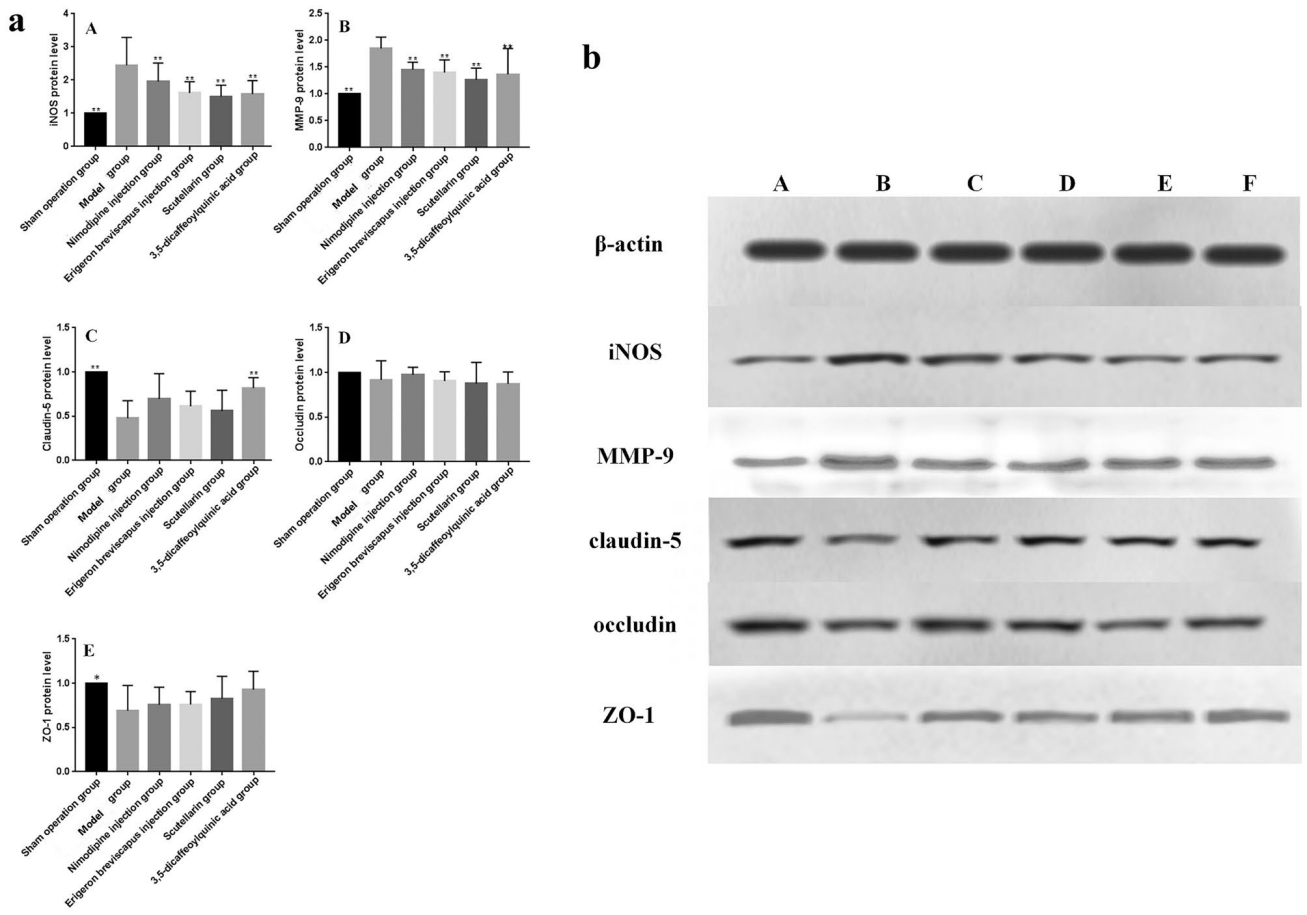
(**a**) Effects of drugs on the expression of iNOS, MMP-9, claudin-5, occludin and ZO-1protein levels in the ischemic brain tissue of MCAO rats. (A-D) represents protein level analysis of expression of iNOS, MMP-9, claudin-5, occludin and ZO-1. The values are presented as mean ± SD. (n = 6), *compared with the model group, *P < 0.05 and **P < 0.01. (**b**) Immunoblotting electrophoresis map of the ROS/RNS-MMPs-TJs pathway. Related indicators in the ischemic brain tissue of rats. Sham operation group (**A**), model group (**B**), Nimodipine injection group (**C**), *E. breviscapus* injection group (**D**), Scutellarin group (**E**) and 3,5-dicaffeoylquinic acid group (**F**).

The expression of iNOS protein in the ischemic brain tissue of rats in our model group, compared with sham operation group, increased significantly (*P* < 0.01). In addition, the expression level of iNOS protein in the ischemic brain tissue of rats in the Nimodipine injection, *E. breviscapus* injection, Scutellarin and 3,5-dicaffeoylquinic acid groups, compared with our model group, was significantly lower (*P* < 0.01), as shown in the Table 6 and Fig. 13.

**Table 6:** Effects of drugs on the expression of iNOS, MMP-9, claudin-5, occludin and ZO-1protein levels in the ischemic brain tissue of MCAO rats.

Group	Dose	n	iNOS		MMP-9		Claudin-5		Occludin		ZO-1	
			$\overline{x} \pm s$	*P* value	$\overline{x} \pm s$	*P* value	$\overline{x} \pm s$	*P* value	$\overline{x} \pm s$	*P* value	$\overline{x} \pm s$	*P* value
Sham operation group	–	6	1.00 ± 0.00	0.000**	1.00 ± 0.00	0.000**	1.00 ± 0.00	0.000**	1.00 ± 0.00	0.357	1.00 ± 0.00	0.013*
Model group	–	6	2.45 ± 0.84	–	1.85 ± 0.21	–	0.48 ± 0.20	–	0.92 ± 0.21	–	0.69 ± 0.29	–
Nimodipine injection group	0.5 mg/kg	6	1.96 ± 0.56	0.092	1.45 ± 0.14	0.013*	0.70 ± 0.28	0.056	0.97 ± 0.09	0.533	0.76 ± 0.20	0.565
*E. breviscapus* injection group	5 mL/kg/d	6	1.61 ± 0.34	0.006**	1.40 ± 0.23	0.005**	0.61 ± 0.17	0.234	0.91 ± 0.10	0.880	0.76 ± 0.15	0.556
Scutellarin group	1.1505 mg/kg	6	1.51 ± 0.34	0.002**	1.27 ± 0.21	0.000**	0.56 ± 0.23	0.463	0.88 ± 0.24	0.636	0.83 ± 0.25	0.254
3,5-Dicaffeoylquinic acid group	0.2335 mg/kg	6	1.58 ± 0.40	0.004**	1.37 ± 0.48	0.003**	0.82 ± 0.12	0.004**	0.87 ± 0.13	0.610	0.93 ± 0.21	0.052

##### Expression of MMP-9 protein

The expression of MMP-9 protein in the ischemic brain tissue of our model group, compared with sham operation group, increased significantly (*P* < 0.01). And, compared with the model group, the expression of MMP-9 protein in the ischemic brain tissue of rats in the Nimodipine injection, *E. breviscapus* injection, Scutellarin and 3,5-dicaffeoylquinic acid groups was significantly lower (*P* < 0.01), as shown in the Table 6 and Fig. 13.

##### Expression of claudin-5 protein

The expression of claudin-5 protein in the ischemic brain tissue of our model group, compared with the sham operation group, was significantly lower (*P* < 0.01). And, compared with the model group, claudin-5 protein expression in the ischemic brain tissue of rats in the Nimodipine injection, *E. breviscapus* injection and Scutellarin groups increased (*P* > 0.05), while claudin-5 protein expression in the ischemic brain tissue of rats in the 3,5-dicaffeoylquinic acid group increased significantly (*P* < 0.01), as shown in the Table 6 and Fig. 13.

##### Expression of occludin protein

Occludin protein expression in the ischemic brain tissue of our model group, compared with the sham operation group, tended to decrease (*P* > 0.05). And, compared with the model group, occludin protein expression in the ischemic brain tissue of the Nimodipine injection group tended to increase (*P* > 0.05), while that in the *E. breviscapus* injection, Scutellarin and 3,5-dicaffeoylquinic acid groups tended to decrease (*P* > 0.05), as shown in the Table 6 and Fig. 13.

##### Expression of ZO-1 protein

The expression of ZO-1 protein in the ischemic brain tissue of our model group, compared with the sham operation group, was significantly lower (*P* < 0.01). And, compared with the model group, the expression of ZO-1 protein in the ischemic brain tissue of rats in the Nimodipine injection, *E. breviscapus* injection, Scutellarin and 3,5-dicaffeoylquinic acid groups increased (*P* > 0.05), as shown in the Table 6 and Fig. 13.

## Conclusion and discussion

Based on the research of network pharmacology, the target prediction results of *E. breviscapus* (Vant.) Hand-Mazz. for the treatment of stroke are similar to the results of literature review. Construct a network diagram of chemical composition-target-ischemic stroke through network pharmacology technology. Through GO analysis and KEGG enrichment analysis, it is found that the treatment of stroke with *E. breviscapus* is related to the neuroprotection mediated by the PI3K-Akt signaling pathway, which in turn affects the blood–brain barrier function, so we chose the blood–brain barrier-related MMP-9, claudin-5, iNOS, occludin and ZO-1 proteins are used for research. It has laid a foundation for the experimental study of the mechanism of the effective components of *E. breviscapus* in the treatment of cerebral ischemia.

*Erigeron breviscapus* injection, Scutellarin and 3,5-dicaffeoylquinic acid have obvious protective effects on brain injury and neurobehavioral abnormalities caused by cerebral ischemia. In addition, they can also reduce the neuronal damage caused by cerebral ischemia and the permeability of BBB.

The results of our research show that *E. breviscapus* injection decreased the expression of iNOS in the ischemic brain tissue of rats caused by cerebral ischemia–reperfusion, but significantly decreased the expression of iNOS in their ischemic brain tissue; and decreased the expression of MMP-9, but increased the expression of claudin-5 and decreased the expression of occludin. We also observed that the Udin gene expression level decreased, but that the occludin protein expression level was not significantly increased and the large ZO-1 gene expression and protein expression levels increased. *Erigeron breviscapus* injection can inhibit the neurotoxicity of ischemic brain tissue in MCAO rats and induce the expression of iNOS mRNA and protein in free radicals (such as mass NO production)^69,70^. It can also reduce the expression level of MMP-9^71–77^, which is closely related to cerebral ischemia–reperfusion injury in MMPs^78,79^. In a word, *E. breviscapus* injection can protect BBB injury induced by cerebral ischemia. The possible mechanism of injury is to inhibit the expression of iNOS and decrease the transcriptional synthesis of MMP-9. Scutellarin decreased the expression of iNOS in the ischemic brain tissue of rats caused by cerebral ischemia–reperfusion, but decreased the expression of iNOS in their ischemic brain tissue. It also decreased the expression of MMP-9, but decreased the expression of MMP-9; had no effect on the expression of claudin-5 and had no effect on the expression of claudin-5 protein. There was an upward trend in the level of occludin gene and protein expression, but no significant increase in the level of ZO-1 gene expression. Scutellarin can inhibit the expression of iNOS mRNA and protein in the ischemic brain tissue of MCAO rats, which has a neurotoxic effect and can induce free radical production (such as mass NO production)^69,70^; reduce the expression level of MMP-9^69–71,78,79^, and gene and protein in MMPs^78,79^, which are closely related to cerebral ischemia–reperfusion injury. More specifically, the possible mechanism of Scutellarin protecting BBB injury induced by cerebral ischemia inhibits iNOS expression and reduces MMP-9 transcriptional synthesis.

Our results show that 3,5-dicaffeoylquinic acid decreased the expression level of iNOS in the ischemic brain tissue of rats caused by cerebral ischemia–reperfusion, and decreased the expression level of iNOS in their ischemic brain tissue. It also decreased the expression level of MMP-9 in the ischemic brain tissue of rats, but significantly decreased their expression of MMP-9; and decreased the expression of claudin-5 in the brain tissue of rats. The expression level of occludin decreased significantly, but the expression level of occludin did not increase significantly, and the expression level of ZO-1 increased significantly. We find that 3,5-dicaffeoylquinic acid can inhibit neurotoxicity in the ischemic brain tissue of MCAO rats, and induce the expression of iNOS mRNA and protein in free radicals (e.g., NO)^69,70^; reduce the expression level of MMP-9^71–77^, mRNA and protein in MMPs^78,79^, which are closely related to cerebral ischemia–reperfusion injury; and up-regulate the closure of the main skeleton protein^80–83^, which constitutes the close link (TJs). More specifically, 3,5-dicaffeoylquinic acid inhibits ROS/RNS production and MMP-9 transcriptional synthesis by reducing the iNOS expression level, and reducing the mechanism of MMP-9 on claudin-5, occludin and ZO-1 degradation to protect BBB injury induced by cerebral ischemia.

*Erigeron breviscapus* injection, Scutellarin and 3,5-dicaffeoylquinic acid exert different therapeutic effects on brain tissue injury, neurobehavioral abnormality, pathological changes of brain tissue, the number of Nissl corpuscle cells, neurons and BBB injury. They increase the permeability of BBB in rats with cerebral ischemia through different mechanisms, so as to protect brain tissue and BBB. At the same time, it is also clear that Scutellarin and 3,5-dicaffeoylquinic acid are one of the pharmacodynamic bases of *E. breviscapus* injection.

RT-PCR and WB were used to detect the expression of the ROS/RNS-MMPs-TJs pathway-related genes and proteins in brain tissues. The expression levels of iNOS and MMP-9 in the ischemic brain tissues of rats increased, while the expression levels of claudin-5, occludin and ZO-1 decreased. These results are consistent with those reported in the literature^70,73,77,83,84^. However, when the expression levels of iNOS, MMP-9, claudin-5, occludin and ZO-1 in brain tissue were detected by RT-PCR, there were significant differences in the expression levels of ZO-1 compared with the sham operation group, but there were no significant differences in the expression levels of iNOS, MMP-9, claudin-5 and occludin, which might be caused by the large standard deviation resulting from the large deviation of the data of the six animals. When S and MMP-9, claudin-5, occludin and ZO-1 protein expression levels were compared with the sham operation group, except the occludin protein expression levels, the iNOS, MMP-9, claudin-5 and ZO-1 protein expression levels were significantly different. In addition, *E. breviscapus* injection, Scutellarin and 3,5-dicaffeoylquinic acid can reduce the expression levels of iNOS and MMP-9 proteins, with significant differences. Our results indicate that 3,5-dicaffeoylquinic acid could also significantly reduce the expression level of claudin-5 protein. There were significant differences in the expression of iNOS, MMP-9, claudin-5 and occludin in some samples, but there was no significant difference in the expression of mRNA. This may be related to data errors caused by the fast degradation of iNOS, MMP-9, claudin-5 and occludin in some samples when RT-PCR was used to detect the indicators.

The molecular mechanism of *E. breviscapus* injection in protecting BBB injury induced by cerebral ischemia in rats was studied through the ROS/RNS-MMPs-TJs signaling pathway. *Erigeron breviscapus* injection contains a variety of chemical constituents. Cell experiments have shown that *E. breviscapus* injection contains 3,5-dicaffeoylquinic acid, 4,5-dicaffeoylquinic acid, 1,5-dicaffeoylquinic acid, 3,4-dicaffeoylquinic acid, caffeic acid, Scutellarin and apigenin. Our research results show that apigenia-O-7-β-d-glucose has protective effects on rat cerebral cortical neurons in vitro. In addition, 1,5-dicaffeoylquinic acid, 1,3-O-dicaffeoylquinic acid, 3,5-dicaffeoylquinic acid and 4,5-dicaffeoylquinic acid can penetrate BBB in vitro to varying degrees. The BBB is composed of vascular endothelial cells and their TJs, the basement membrane of endothelial cell outer junction, and the glial membrane barrier formed by astrocytes under basement membrane around blood vessels. The injury of BBB after cerebral ischemia is caused by many factors. Therefore, the TJs between the vascular endothelial cells and the destruction of TJs are two factors that lead to BBB injury induced by cerebral ischemia. Meanwhile, the flavonoids Scutellarin and caffeoyl 3,5-dicaffeoylquinic acid in *E. breviscapus* injection were selected through our review of the literature and preliminary experiments. Based on the MCAO rat model, the protective effects and molecular mechanism of *E. breviscapus* injection, Scutellarin and 3,5-dicaffeoylquinic acid on BBB injury induced by cerebral ischemia were studied by various methods. In this study, only two components of Scutellarin and 3, 5-dicaffeoylquinic acid were selected to evaluate the effectiveness of cerebral ischemia based on animal experiments, and the pharmacodynamics of other chemical components to protect cerebral ischemia could also be explored, which was more conducive to revealing the pharmacodynamics substance basis of *E. breviscapus* injection in the clinical treatment of ischemic stroke. Some studies have shown that kaempanol can significantly improve the cognitive dysfunction, neurobehavioral abnormalities and pathological damage in rats caused by chronic cerebral ischemia^85^, it also has a neuroprotective effect on focal cerebral ischemia–reperfusion injury^86^. Naringin has a neuroprotective effect on focal ischemia^87,88^, it can reduce the water content, reduce the area of cerebral infarction, reduce the content of MDA, improve the activity of SOD and have a good ability of free radical scavenging^89^. Therefore, more pharmacodynamics material basis can be studied in the future to elucidate more active components of *E. breviscapus.*

In addition to studying the molecular mechanism of *E. breviscapus* injection in protecting BBB injury induced by cerebral ischemia through the ROS/RNS-MMPs-TJs signaling pathway and TJ between vascular endothelial cells, BBB is composed of multiple barriers and there are many factors affecting BBB permeability. In addition, neurovascular units are neurons and their axons, glia cells, vascular endothelial cells, pericytes, basement membrane and cells. External matrix composition^90,91^, the components are coordinated interaction, BBB is the core structure of the neurovascular unit (NVU). Therefore, taking (NVU) as the breakthrough point, the mechanism of *E. breviscapus* injection and other components of *E. breviscapus* injection in treating ischemic stroke was studied deeply from the overall concept of traditional Chinese medicine. In addition, how *E. breviscapus* injection and other components affect the mutual regulation mechanism of each component of a neurovascular unit has been clarified. *E. breviscapus* injection was systematically and comprehensively studied and evaluated. Previous studies have shown that 1,5-dicaffeoylquinic acid, 1,3-dicaffeoylquinic acid, 3,5-dicaffeoylquinic acid and 4,5-dicaffeoylquinic acid in *E. breviscapus* can also pass through a BBB^21^ in vitro. In addition, 3,5-dicaffeoylquinic acid, 4,5-dicaffeoylquinic acid, 1,5-dicaffeoylquinic acid, 3,4-dicaffeoylquinic acid, caffeic acid, Scutellarin, apigenin and Apigenia-O-7-β-D-glucose have protective effects on the survival of rat cerebral cortical neurons in vitro^15–18^. Therefore, the pharmacodynamics of *E. breviscapus* injection such as 5-caffeoylquinic acid, chlorogenic acid, 4-caffeoylquinic acid, caffeic acid, 1,3-dicaffeoylquinic acid, isochlorogenic acid B, breviscapine, 4,5-dicaffeoylquinic acid and other chemical components to protect BBB injury caused by cerebral ischemia are beneficial in revealing the clinical application of *E. breviscapus* injection in the treatment of cerebral ischemia. In this study, based on ROS/ RNS-MMPS-TJS signaling pathway, we investigated the molecular mechanism of *E. breviscapus* injection's protection against cerebral ischemia-induced BBB injury through the tight junction between vascular endothelial cells as the entry point. In addition, more studies should be conducted on the protective mechanism of *E. breviscapus* injection against cerebral ischemia. For example, Xia-min Hu et al.^92^ found that Scutellarin has protective effects for cerebral injury through their study regulating the expression of NOS isoforms and angiogenic molecules. Compared with Xia-min Hu, TTC staining, neurobehavioral scoring, EB method and WB method were adopted in all of us. However, in this study, transmission electron microscopy was also used to study the morphological and structural changes of BBB, and elucidate the protective mechanism of BBB, while their studies showed angiogenic influence on the key molecules, vascular colorectal growth factor (VEGF) and basic fibroblast growth factor were studied, which opened a new idea for the mechanism study of *E. breviscapus* injection in the treatment of cerebral ischemia.

The brain distribution of Scutellarin in *E. breviscapus* injection has also been studied^93^, but no other active ingredients have been found in the brain. Therefore, to study the distribution of effective ingredients and active ingredients of *E. breviscapus* injection in the brain tissue of MCAO model rats, it is necessary to clarify the target of *E. breviscapus* injection and its active ingredients in the treatment of ischemic stroke. This clarification will help reveal the main effective ingredients of *E. breviscapus* injection in the treatment of ischemic stroke, and assist in the exploration of the metabolic components in the brain after administration of *E. breviscapus* injection for the treatment of ischemic stroke. This will provide a scientific theoretical basis for the clinical application of *E. breviscapus* injection.

## Supplementary Information

Supplementary Information 1.

Supplementary Information 2.

## Data Availability

The data generated during this study are available from the corresponding author on reasonable request.

## Abbreviations

BBB: Blood brain barrier
CCA: Common carotid artery
ECA: External carotid artery
EB: Evans blue
HPLC: High performance liquid chromatography
iNOS: Inducible nitric oxide synthase
MCAO: Middle cerebral artery occlusion
MMPs: Martrix metalloproteinase
RT-PCR: Real-time PCR
ROS: Reactive oxygen species
RNS: Reactive nitrogen species
TTC: TTC stainning
TJ: Tight junction
TEM: Transmission electron microscope
WB: Western-blotting
ZO: Zonulae occludens protein
