# Supplementary material for: Protective mechanism of Erigeron breviscapus injection on blood–brain barrier injury induced by cerebral ischemia in rats
Source: Sci Rep. 2021 Sep 16;11:18451. doi: 10.1038/s41598-021-97908-x (PMC8446017; doi:10.1038/s41598-021-97908-x)
Supplement: Supplementary file 1 — Supplementary Information 1. [file 41598_2021_97908_MOESM1_ESM.pdf]

Supplementary Materials

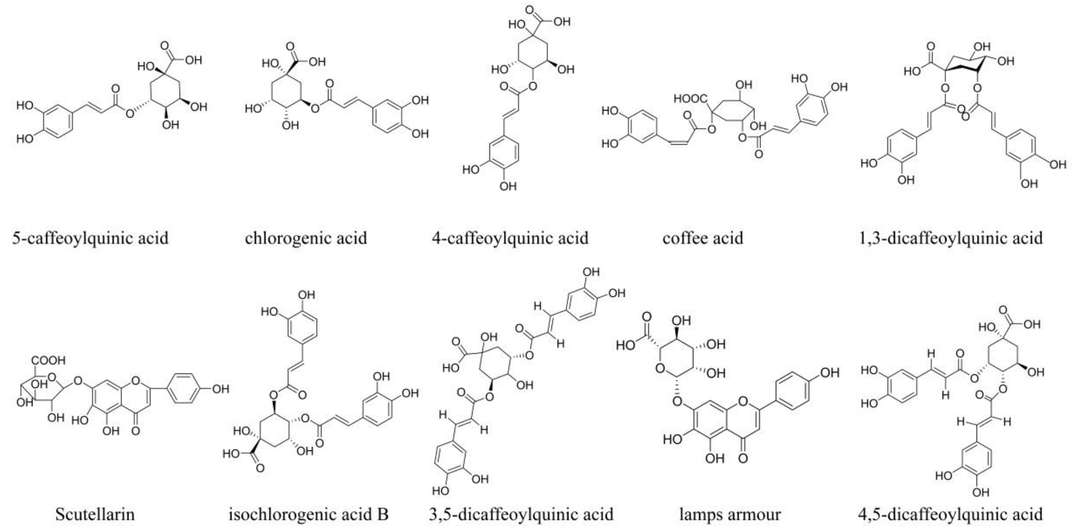

Supplementary figure 1 The chemical structures of the ten components.

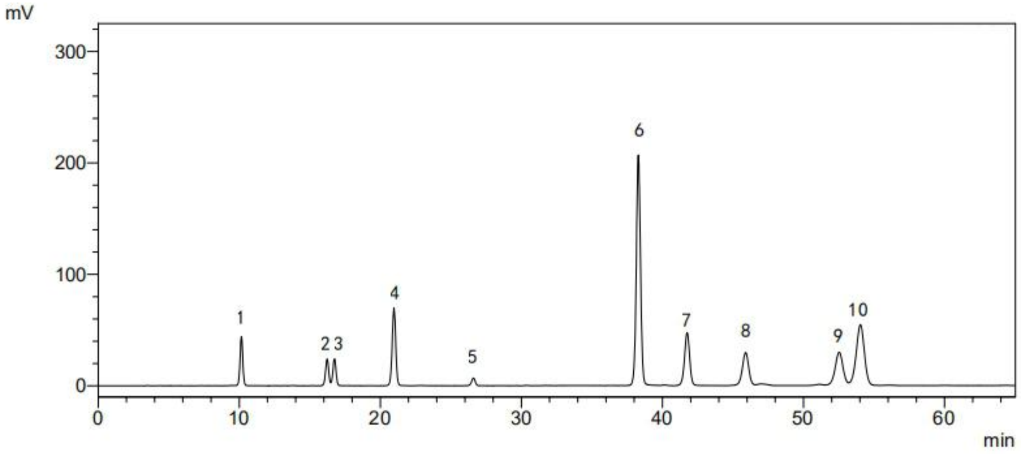

(1 5-caffeoylquinic acid; 2 chlorogenic acid; 3 4-caffeoylquinic acid; 4 coffee acid; 5 1,3-dicaffeoylquinic acid;6 Scutellarin; 7 isochlorogenic acid B; 8 3,5-dicaffeoylquinic acid; 9 lamps armour; 10 4,5-dicaffeoylquinic acid)

Supplementary figure 2 HPLC analysis of the test solution

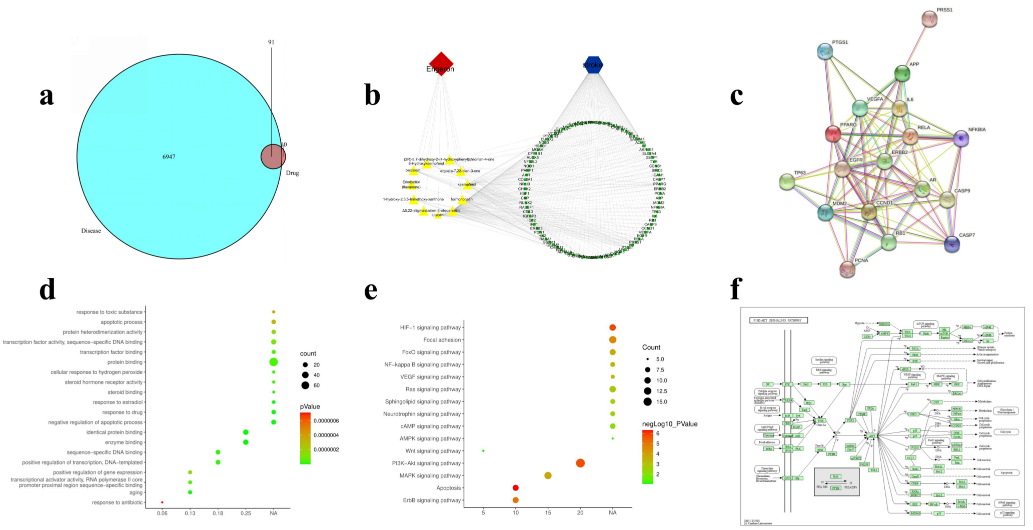

Supplementary figure 3 Network pharmacological study on cerebral ischemia treated by *Erigeron breviscapus*.(a)Drug target-disease target related target map.(b)The construction and analysis diagram of the chemical composition-target-ischemic stroke interactive network.(c)Target gene interaction mapping.(d)GO enrichment analysis.(e)KEGG enrichment analyses.(f)“PI3K-Akt signaling pathway”signal path diagram.

Supplementary table 1 Gradient elution program

Number	Time(min)	A(0.4% phosphoric acid, %)	B(acetonitrile，%)
1	0	90	10
2	25	83	17
3	26	80	20
4	65	80	20

Supplementary table 2 Effective chemical constituents of *Erigeron breviscapus*

Mol ID	Molecule Name	OB (%)	DL
MOL000006	luteolin	36.16	0.25
MOL000098	quercetin	46.43	0.28
MOL000392	formononetin	69.67	0.21
MOL000422	kaempferol	41.88	0.24
MOL000816	ergosta-7,22-dien-3-one	44.88	0.72
MOL001040	(2R)-5,7-dihydroxy-2-(4-hydroxyphenyl)chroman-4-one	42.36	0.21
MOL002712	6-Hydroxykaempferol	62.13	0.27
MOL002714	baicalein	33.52	0.21
MOL002914	Eriodyctiol (flavanone)	41.35	0.24
MOL007963	1-hydroxy-2,3,5-trimethoxy-xanthone	101.06	0.30
MOL007984	Δ5,22-stigmastadien-3-ol	43.83	0.76

Supplementary table 3 Related targets of *Erigeron breviscapus* for potential treatment of ischemic stroke

Gene Name	Gene Name	Gene Name	Gene Name
PTGS1	AR	PRSS1	RELA
EGFR	VEGFA	CCND1	CASP9
RB1	IL6	TP63	NFKBIA
MDM2	APP	PCNA	ERBB2
PPARG	CASP7	ICAM1	BIRC5
CCNB1	TYR	GSTP1	SLC2A4
AKR1B1	F7	ACHE	GABRA1
BCL2	FOS	PLAU	AHSA1
CASP3	ELK1	POR	CASP8
RAF1	PRKCA	HIF1A	RUNX1T1
ACACA	CYP3A4	CAV1	MYC
CYP1A1	SELE	VCAM1	PTGER3
DUOX2	NOS3	HSPB1	MGAM
CYP1B1	ALOX5	NFE2L2	NQO1
PARP1	AHR	COL3A1	NR1I3
CHEK2	HSF1	CRP	RUNX2
RASSF1	CTSD	IGFBP3	IGF2
IRF1	ERBB3	PON1	HK2
RASA1	GSTM1	GSTM2	CHRM1
ESR1	ADRA1A	ESR2	GSK3B
CHEK1	PGR	CHRM2	IKBKB
MAPK8	AKR1C3	NR3C2	NR3C1
CYCS	NOX5	APOD	
