# Supplementary material for: Protective mechanism of Erigeron breviscapus injection on blood–brain barrier injury induced by cerebral ischemia in rats
Source: Sci Rep. 2021 Sep 16;11:18451. doi: 10.1038/s41598-021-97908-x (PMC8446017; doi:10.1038/s41598-021-97908-x)

# Protective mechanism of *Erigeron breviscapus* injection on blood-brain barrier injury induced by cerebral ischemia in rats

***Guangli Liu[1,2], Yan Liang [1],Min Xu[1], Ming Sun [1], Weijun Sun [1], You Zhou [1], Xiaojuan Huang [3], Wenjie Song [3], Yuan Liang [1],Zhang Wang [3*]***

*1.College of Pharmacy, Chengdu University of Traditional Chinese Medicine, Chengdu, Sichuan,611137,China;*

*2.Hospital Pharmaceutical Department, Xuzhou Maternity and Child Health Care Hospital,Xuzhou ,Jiangsu,221000,China;*

*3.College of Ethnomedicine, Chengdu University of Traditional Chinese Medicine, Chengdu, Sichuan,611137, China*

**Correspondence: Zhang Wang, E-mail: wangzhangcqcd@cdutcm.edu.cn, Tel:+86 28 61656141.*

β-actin

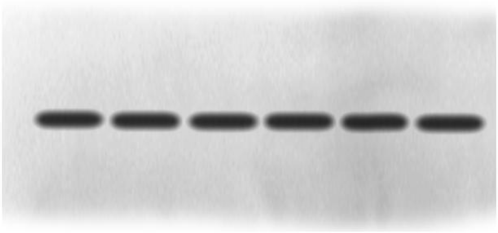

iNOS

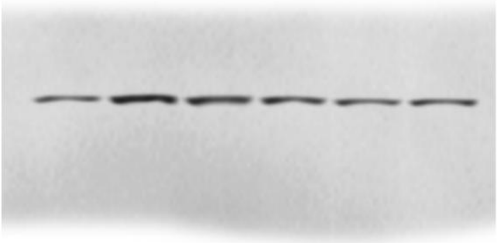

MMP-9

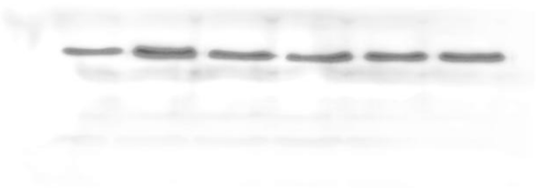

claudin-5

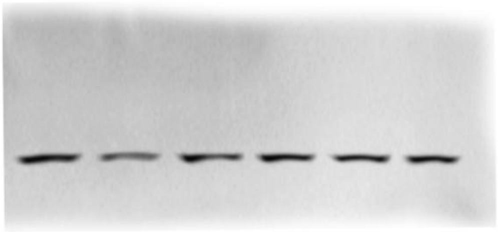

occludin

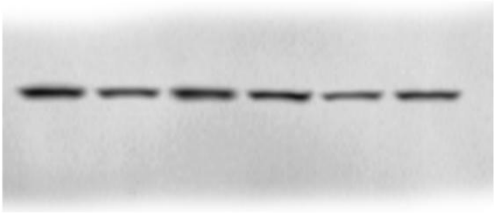

ZO-1

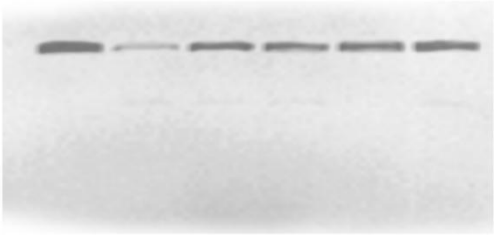

Supplement: Supplementary file 2 — Supplementary Information 2. [file 41598_2021_97908_MOESM2_ESM.pdf]
